# MDC1 counteracts replication fork reversal and mediates chemosensitivity in BRCA1/2-deficient tumors

**DOI:** 10.1038/s41388-025-03659-8

**Published:** 2025-12-17

**Authors:** Hülya Dogan, Martin Liptay, Joana S. Barbosa, Ewa Gogola, Alexandra A. Duarte, Jonas A. Schmid, Ismar Klebic, Merve Mutlu, Myriam Siffert, Paola Francica, Israel Salguero, Marieke van de Ven, Renske de Korte-Grimmerink, Stephen P. Jackson, Jos Jonkers, Massimo Lopes, Diego Dibitetto, Sven Rottenberg

**Affiliations:** 1 Institute of Animal Pathology, Vetsuisse Faculty, University of Bern, Bern, 3012 Switzerland; 2 Division of Molecular Pathology, The Netherlands Cancer Institute, Amsterdam, 1066CX The Netherlands; 3 Institute of Molecular Cancer Research, University of Zurich, Zurich, Switzerland; 4 Wellcome/Cancer Research UK Gurdon Institute, University of Cambridge, Tennis Court Rd, Cambridge, CB2 1QN UK; 5 Mouse Clinic for Cancer and Aging Research (MCCA), Preclinical Intervention Unit, The Netherlands Cancer Institute, Amsterdam, 1066CX The Netherlands; 6 Cancer Research UK Cambridge Institute, University of Cambridge, Robinson Way, Cambridge, CB2 0RE UK; 7 Oncode Institute, The Netherlands Cancer Institute, Amsterdam, 1066CX The Netherlands; 8 Bern Center for Precision Medicine, Department for BioMedical Research, University of Bern, Bern, 3012 Switzerland; 9 Present Address: Spanish National Cancer Research Center, Madrid, 28029 Spain; 10 Present Address: Department of Experimental Oncology, Istituto di Ricerche Farmacologiche Mario Negri IRCCS, Via Mario Negri, 2, Milan, 20156 Italy

**Keywords:** Breast cancer, DNA damage response, Cancer therapeutic resistance

## Abstract

MDC1 is a key protein in DNA damage signaling. When DNA double-strand breaks (DSBs) occur, MDC1 localizes to the sites of DNA damage to promote the recruitment of other factors, including the 53BP1-mediated DSB repair pathway. By studying mechanisms of poly (ADP-ribose) polymerase inhibitor (PARPi) resistance in BRCA2; p53-deficient mouse mammary tumors, we identified a thus far unknown role of MDC1 in replication fork biology. Our results show that MDC1 localizes at active replication forks during normal DNA replication and regulates replication fork progression. It suppresses spontaneous fork reversal and regulates fork nucleolytic processing thereby promoting sensitivity to PARPi and cisplatin. In this way, MDC1 loss improves DNA damage tolerance and causes chemoresistance in BRCA1/2-deficient cells. We demonstrate that limiting MRE11 activity abolishes the reduced fork speed while MRE11 inhibition/depletion overcomes PARPi resistance in these cells. Overall, our data provides new insights into the role of MDC1 in replication fork progression that mediates PARPi- and cisplatin-induced DNA damage, in addition to its role in DSB repair.

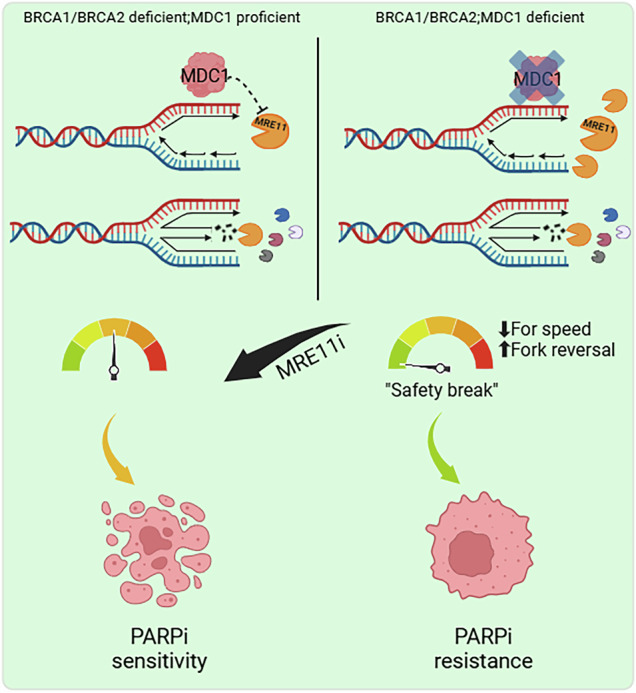

## Introduction

Despite the success of poly(ADP-ribose) polymerase (PARP) inhibitors in treatment of patients with BRCA1- or BRCA2-deficient tumors [1–3] long-lasting clinical response rates in patients with advanced disease are limited by the development of resistance. Regrettably the underlying mechanisms of PARPi resistance in patients, have not been fully elucidated. Using *K14cre;Brca1*^*F/F*^*;Trp53*^*F/F*^ (KB1P) and *K14cre;Brca2*^*F/F*^*;Trp53*^*F/F*^ (KB2P) genetically engineered mouse models of hereditary breast cancer [4, 5], we have identified various BRCA1- or BRCA2-independent mechanisms of PARPi resistance [reviewed in [6, 7]]. In stark contrast to KB1P tumors [8, 9], in none of the PARPi-resistant KB2P BRCA2-deficient tumors we found evidence for homologous recombination (HR) restoration. Instead, we found that a significant fraction of PARPi-resistant KB2P tumors showed homozygous loss of the poly (ADP-ribose) glycohydrolase (*Parg*) gene [6]. Another resistance mechanism identified in the KB2P model is the protection of replication fork (RF) stability [10], and we recently showed that loss of *H2afx* gene expression is an underlying mechanism [11].

Regarding the effect of PARPi on RF biology, it has been previously reported that RFs, which are remodeled upon DNA damage and mild replication stress, are maintained in the reversed state by transient PARP-mediated inhibitory ADP ribosylation of the RECQ1 helicase [12, 13]. PARP1 therefore acts as a molecular switch to control transient fork reversal and RF restart following genotoxic stress [13]. While untreated cells gain extra time to repair DNA damage through RF reversal, PARPi-treated cells are unable to efficiently maintain forks in a reversed state, resulting in increased DNA breakage and downstream requirement for HR-mediated DSB repair. Furthermore, the group of Jiri Bartek reported that PARPi increases the speed of RF elongation by interfering with the PARP1-p53-p21-fork speed-regulatory network [14]. This suggests that alterations of RF biology may affect the success of PARP inhibition.

Here we show that MDC1 is an important mediator of the cellular response to PARPi and cisplatin, an effect we link to its participation in replication fork reversal. PARPi-induced uncontrolled fork restart and increased speed of RF progression are counteracted by MDC1 loss, leading to PARPi resistance in BRCA2; p53-deficient mammary tumors. In line with this model, MDC1 loss also causes PARPi resistance in BRCA1-deficient tumor cells without restoring RAD51 foci formation, a typical hallmark of HR restoration that we frequently found in PARPi-resistant KB1P tumors harboring mutations in the 53BP1-RIF1-REV7-CST-Shieldin pathway [15–18]. Hence, our work reveals a previously unknown function of MDC1 in RF biology, a role that contributes to DNA damage induced by genotoxic agents and the response of BRCA1- or BRCA2-deficient tumors to clinically relevant anti-cancer drugs, such as PARPi and cisplatin.

## Materials and methods

### Materials availability

All unique/stable reagents generated in this study will be made available on request, but we may require a payment and/or a completed Materials Transfer Agreement if there is potential for commercial application.

### Mice

The Animal Ethics Committee of The Netherlands Cancer Institute (Amsterdam, the Netherlands) and the Animal Ethics Committee of the Canton of Bern (Switzerland, Application number BE40/18) approved all animal experiments. All experiments were performed in accordance with the Dutch Act on Animal Experimentation (November 2014) and the Swiss Act on Animal Experimentation (December 2015). CRISPR-Cas9-modified organoids lines derived from *K14cre;Brca2*^*F/F*^*;Trp53*^*F/F*^ (KB1P) female mice were transplanted in 6–9 weeks-old NMRI nude mice for the in vivo validation.

### Cell lines

The KB2P1.21 and KB2P3.4 cell lines were previously established from a *K14cre;Brca2*^*F/F*^*;Trp53*^*F/F*^ (KB2P) mouse mammary tumor as described by Evers et al. [19]. The KB2P1.21R cell line was derived from the KB2P1.21 cells by reintroducing BRCA2 as described by Evers et al. [20]. The KB1P-G3 cell line was previously established from a KB1P mouse mammary tumor and cultured as described by Jaspers et al. [17]. The RPE-1-hTERT *MDC1* knockout cell line and RPE-1-hTERT FRT-derived cell lines stably expressing inducible GFP-tagged constructs were established and previously described by Salguero et al. [21]. The *BRCA1;TP53*^-/-^ RPE-1 cell line was established and previously described by Zimmermann et al. [22]. All the KB1P and KB2P lines were grown in Dulbecco’s Modified Eagle Medium/Nutrient Mixture F-12 (DMEM/F12; Gibco) supplemented with 10% fetal calf serum (FCS, Sigma Aldrich), 50 units/ml penicillin-streptomycin (Gibco), 5 µg/ml Insulin (#I0516, Sigma Aldrich), 5 ng/ml cholera toxin (#C8052, Sigma Aldrich) and 5 ng/ml murine epidermal growth-factor (EGF, #E4127, Sigma Aldrich). The PEO1 and HEK293FT cell line (RRID:CVCL_6911) was cultured in DMEM-high glucose with sodium bicarbonate, ʟ-glutamine and sodium pyruvate (Sigma Aldrich) supplemented with 10% fetal calf serum (FCS, Sigma Aldrich) and 50 units/ml penicillin-streptomycin (Gibco). All the RPE-1-hTERT cell lines were cultured in DMEM/F12 (Gibco) supplemented with 10% FCS, 100 U/ml penicillin, 100 ng/ml streptomycin, 17 ml NaHCO_3_ 7.5% per 500 ml (Sigma Aldrich) and 2 mM L-glutamine. In addition, medium of the RPE-1-hTERT FRT derived cells expressing GFP-tagged MDC1 constructs contained 0.5 mg/ml G418 disulfate salt solution (#G8168, Sigma Aldrich). Expression of the constructs was induced by adding 0.5 µg/ml of doxycycline in the growth medium 48 h prior experiments. The cells were maintained in the presence of doxycycline for the whole duration of the experiment.

Mouse mammary tumor-derived KB2P and KB1P cell lines and the human *BRCA1;TP53*^-/-^ RPE-1-hTERT cell lines were grown in low oxygen atmosphere at standard temperature (37 °C, 3% O_2_, 5% CO_2_). HEK293FT, PEO1 and RPE-1-hTERT cell lines were grown in standard conditions (37 °C, 5% O_2_ 5% CO_2_). Testing for mycoplasma contamination was performed on a regular basis.

### Tumor-derived organoids

The KB2P26S.1 3D tumor organoid line was previously established from a *Brca2*^*-/-*^*;Trp53*^*-/-*^ mouse mammary tumor and cultured as described by Duarte et al. [23]. Briefly, cultures were embedded in Culturex Reduced Growth Factor Basement Membrane Extract Type 2 (BME, Trevigen; 40 μl BME:growth media 1:1 drop in a single well of 24-well plate) and grown in Advanced DMEM/F12 (AdDMEM/F12, Gibco) supplemented with 1 M HEPES (Sigma Aldrich), GlutaMAX (Gibco) 50 units/ml penicillin-streptomycin (Gibco), B27 (Gibco), 125 μM N-acetyl-L-cysteine (Sigma Aldrich) and 50 ng/ml murine epidermal growth factor (Sigma Aldrich). Organoids were cultured under standard conditions (37 °C, 5% CO_2_) and regularly tested for mycoplasma contamination. Further in vitro culture details and gene editing details are provided in the “method” details section.

### CRISPR/Cas9-based genetic screen

The PARPi resistance screens were performed in the KB2P3.4 tumor cell line, which was previously established from a KB2P tumor (Evers et al. [19]). Mouse GECKO v2 library, pool B (62,804 gRNAs targeting 20,628 genes (3 gRNAs/gene) and including 1000 control non-targeting gRNAs), was stably introduced into the cells by lentiviral transduction at the multiplicity of infection (MOI) of 1.5. 6 independent transductions were carried out to obtain mutagenized cells for biological replicates of the PARPi resistance screen. To perform the genetic screen at 50× library coverage, 3 × 10^6^ mutagenized KB2P3.4 cells in each replicate were plated in 10-cm flasks, at low density (30,000 cells per flask) and grown in the medium containing 200–300 nM AZD2461 for 3 weeks. The medium with the PARPi was refreshed twice a week. Cells were harvested before and after PARPi treatment for genomic DNA isolation. Subsequently, gRNA sequences were amplified from genomic DNA by two rounds of PCR amplification as described previously [24, 25]. Resulting PCR products were purified using MinElute PCR Purification Kit (Qiagen) and submitted for Illumina sequencing. Sequence alignment and enrichment analysis (day 0 vs PARPi-treated population) was carried out using MAGeCK software [26].

### Gene editing, silencing, plasmids and cloning

Lentiviral stocks were generated by transient transfection of HEK293FT cells. On day 0, 6 × 10^6^ HEK293FT cells were seeded in 150 cm cell culture dishes and on the next day transiently transfected with lentiviral packaging plasmids and the pGS-Cas9 (Neo) or iKRUNC-Puro/plentiCRISPRv2 vector containing the respective gRNA or a non-targeting gRNA using 2x HBS (280 nM NaCl, 100 mM HEPES, 1.5 mM Na_2_HPO_4_, pH 7.22), 2.5 M CaCl_2_ and 0.1x TE buffer (10 mM Tris pH 8.0, 1 mM EDTA pH 8.0, diluted 1:10 with dH_2_O). After 30 h, virus-containing supernatant was concentrated by ultracentrifugation at 20,000 rpm for 2 h in a SW40 rotor and the virus was finally resuspended in 100 μl PBS. The virus titer was determined using a qPCR Lentivirus Titration Kit (#LV900, Applied Biological Materials). For lentiviral transduction, 150,000 target cells were seeded in 6-well plates. 24 h later, virus at the MOI of 50 was applied with 8 μg/ml Polybrene (Merck Millipore). Virus-containing medium was replaced with medium containing puromycin (3.5 μg/ml, Gibco) 24 h later. Puromycin selection was performed for 3 days; subsequently cells were expanded and frozen down at early passage. Tumor-derived organoids were transduced according to a previously established protocol [23]. The target sites modifications of the polyclonal cell pools were analyzed by TIDE analysis which is described below.

For CRISPR/Cas9-mediated genome editing, we used the iKRUNC system described previously [27], KB2P1.21 and KB2P3.4 cells, or KB2P26S.1 organoids were first transduced with the lentiviral pGS-Cas9 (Neo) construct and grown under G418 selection (500 μg/ml) for 5 days. Next, neomycin-selected cells were incubated with lentiviral supernatants of iKRUNC-Puro vectors containing the respective gRNA or a non-targeting gRNA and exposed to 3 μg/ml puromycin for 5 days. To induce gRNA expression, puromycin-surviving cells were treated for another 5 days with 3 μg/ml doxycycline (Sigma Aldrich). For CRISPR/Cas9-mediated genome editing with lentiCRISPRv2 system, KB1P-G3 cells were transduced with the pLentiCRISPRv2 vector encoding non-targeting gRNA, *Mdc1*-targeting gRNA1 or *Mdc1*-targeting gRNA2. The cells were then grown under Puromycin (3 μg/ml) selection for 5 days. CRISPR gRNA sequences for modification of *Mdc1* were chosen from the GeCKo library v2 [24]. The gRNA sequences were as follows: *Mdc1* gRNA1: 5′-GGTGTGTGGCGAATGGACAA-3′ targeting exon 4; *Mdc1* gRNA2: 5′-TTCGCCACACACCTTCCAGA-3′ targeting exon 4; Non-targeting (NT) gRNA: 5′TGATTGGGGGTCGTTCGCCA-3′. For CRISPR/Cas9-mediated genome editing of the *BRCA1;TP53*^-/-^ RPE-1-hTERT cells, cells were transduced with the pX330 vector encoding non-targeting gRNA or *MDC1*-targeting gRNA. The cells were then grown under Puromycin (3 μg/ml) selection for 5 days. All constructs sequences were verified by Sanger sequencing. *MDC1* gRNA: 5′-CACCTCGGGAAGAATGTGGT -3′; Non-targeting (NT) gRNA: 5′-TGATTGGGGGTCGTTCGCCA-3′. To assess the modification rate at the gRNA-targeted region of *Mdc1*/*MDC1*, cells were pelleted and genomic DNA was extracted using the QIAmp DNA mini kit (Qiagen) according to manufacturer’s protocol. Target loci were amplified using Phusion High Fidelity Polymerase (Thermo Scientific) using a 3-step protocol. For amplification of the gRNA target sites in mouse cells:(1) 98 °C for 30 s, (2) 35 cycles at 95 °C for 15 s, 55 °C for 15 s and 72 °C for 30 s, (3) 72 °C for 7 min. For amplification of the gRNA target sites in human cells: (1) 98 °C for 30 s, (2) 35 cycles at 95 °C for 15 s, 65 °C for 15 s and 72 °C for 15 s, (3) 72 °C for 7 min. Reaction mix consisted of 10 μl of 2x Phusion Mastermix (Thermo Fisher), 1 μl of 10 μM forward and reverse primer and 100 ng of DNA in 20 μl total volume. PCR products were purified using the QIAquick PCR purification kit (Qiagen) according to manufacturer’s protocol and submitted with corresponding forward primers for Sanger sequencing to confirm target modifications using the TIDE algorithm [28].

For siRNA transfections, ON-TARGETplus siRNA SMARTpools (Dharmacon) for mouse *Mre11, RecQ1* or *Smarcal1* and ON-TARGETplus non-targeting siRNA were transfected into the cells using Lipofectamine RNAiMAX (Invitrogen) according to the manufacturer’s instructions. All experiments were carried out between 48 and 72 h post-transfection.

MISSION shRNA plasmids were purchased from Sigma. Non-targeting shRNA control and shRNA targeting human MDC1 (XM_376479) was used to produce lentiviral particles to generate stable knockdown cells.

### Clonogenic assays

To assess the growth and survival upon exposure to PARPi, KB2P1.21, KB2P3.4 or KB1P-G3 cells were seeded in 6-well plates in the following densities: 5000 cells/well (KB2P1.21 and KB2P3.4) and 4500 cells/well (KB1P-G3). 7000 cells/well was plated for PEO1 cells in 12-well plate. Clonogenic assays with RPE-1 cells were performed by plating 500 cells in 10-cm dish. The treatment of cells with DMSO or indicated concentrations of PARPi, olaparib or AZD2461 started at the day of plating the cells and lasted for the whole duration of the experiment. The medium with DMSO or PARPi was refreshed twice a week. In the clonogenic assays with cisplatin, cells were grown in the cisplatin-containing medium for the whole duration of the experiment and the medium was refreshed twice a week. The control, DMSO-treated plates were fixed 7 days (mouse cells) or 10 days (RPE-1 cells) after seeding. The PARPi- or cisplatin-treated plates were fixed after 10 (mouse cells, PARPi) or 14 (mouse cells, cisplatin; RPE-1 cells, PARPi) days. The fixation was done with 4% formalin and the surviving colonies stained with 0.1% crystal violet. The survival and growth of mouse cells was analyzed in an automated manner using the ImageJ ColonyArea plugin described previously [29]. The survival of RPE-1 cells was assessed by manual counting of colonies. For the competition assays, cells were collected before and after the experiment for gDNA isolation and TIDE analysis as described above. For the clonogenic assay with combined olaparib and mirin treatment, 5000 KB2P1.21 cells were seeded in 6 well plates 1 day before start of the treatment. Next day, medium containing the indicated concentrations of DMSO, olaparib/DMSO, mirin/DMSO or olaparib/mirin was added to the cells. For clonogenic assay with siRNA-mediated knockdown cells were seeded after 48 h post-transfection. Medium with drugs was refreshed twice a week. The next steps were carried out as described above.

### CTB proliferation assay

To analyze the proliferation rate of control (non-targeting gRNA) or *Mdc1*-modified KB2P1.21 cells, 1000 cells were seeded in 96-well plates 1 day before the experiment. Rate of proliferation was analyzed using CellTiter-Blue® Cell Viability Assay (Promega) following manufacturer’s instructions. Fluorescence intensity of culture medium upon incubation with CellTiter-Blue reagent was measured on four consecutive days.

### In vivo studies

**Fig. 1:**
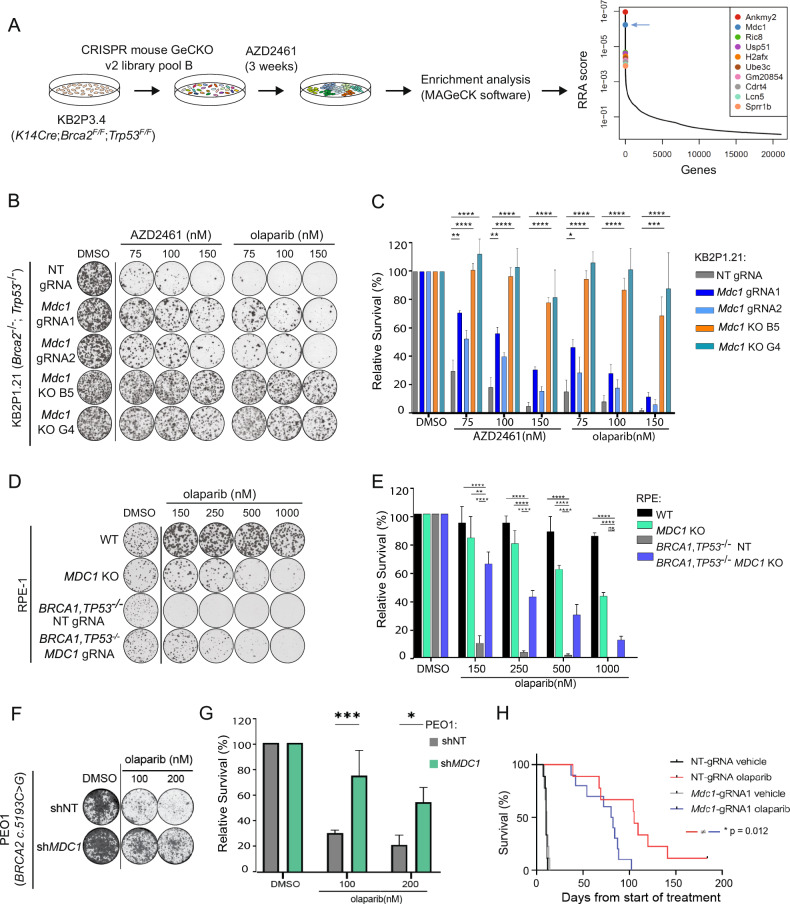
Loss of MDC1 promotes PARPi resistance in vitro and in vivo*.* **A** A schematic overview of the genome-wide loss of function genetic screens. gRNAs are ranked based on *P* values and robust rank aggregation (RRA) algorithm. **B** Representative images and **C** quantification of clonogenic assays with KB2P1.21 cells treated with PARP inhibitors or DMSO. The data represent mean ± SEM of three independent experiments. Two-way ANOVA followed by Dunnet’s test was performed; *p < 0.05, **p < 0.01, ***p < 0.001, ****p < 0.0001. **D** Representative images and **E** quantification of clonogenic assays with RPE-1 cells treated with olaparib or DMSO. The data represent mean ± SEM of three independent experiments. Two-way ANOVA followed by Dunnet’s test was performed; *p < 0.05, **p < 0.01, ***p < 0.001, ****p < 0.0001. **F** Representative images and **G** quantification of clonogenic assays with PEO1 cells treated with PARP inhibitor or DMSO. The data represent mean ± SEM of three independent experiments. Two-way ANOVA followed by Dunnet’s test was performed; *p < 0.05, ***p < 0.001. **H** Kaplan-Meier curve showing the overall survival of the vehicle**-** or olaparib-treated mice. *P* value was calculated with the Mantel-Cox test.

For tumor organoid transplantation, organoids were collected, incubated with TripLE at 37 °C for 5 min, dissociated into single cells, washed in PBS, resuspended in tumor organoid medium and mixed in a 1:1 ratio of tumor organoid suspension and BME. Organoid suspension containing a total of 5 × 10^4^ cells were injected in the fourth right mammary fat pad of 6–9 week-old NMRI nude mice. Mammary tumor size was measured by caliper on at least 3 days per week and tumor volume was calculated (length × width^2^/2). Treatment of tumor bearing mice was initiated when tumors reached a size of ~75 mm^3^, at which point mice were separated into two vehicle-treated groups (NT gRNA n = 5, *Mdc1*-targeting gRNA1 n = 5) and AZD2461-treated groups (NT gRNA n = 5, *Mdc1*-targeting gRNA1 n = 5). AZD2461 (100 mg/kg) was administered orally for 28 consecutive days, control mice were dosed with vehicle only. Animals were anesthetized with isoflurane, sacrificed with CO_2_ followed by cervical dislocation when the tumor reached a volume of 1500 mm^3^. Tumor sampling included cryopreserved tumor pieces, fresh frozen tissue and formalin-fixed material (4% (v/v) formaldehyde in PBS). For the in vivo experiment using olaparib presented in Fig. 1, treatment of tumor bearing mice was initiated when tumors reached a size of ∼150 mm^3^, at which point mice were separated into two untreated/vehicle groups (NT gRNA n = 10, *Mdc1*-targeting gRNA1 n = 10) and olaparib-treated group (NT gRNA n = 10, *Mdc1*-targeting gRNA1 n = 10). Treatment with vehicle/olaparib (100 mg/kg) was administered intraperitoneally for 56 consecutive days. Animals were anesthetized with isoflurane, sacrificed with CO_2_ followed by cervical dislocation when the tumor reached a volume of 1000 mm^3^.

### Immunofluorescence

Cells were seeded on coverslips in 24-well plates 3 days before the experiment. For analysis of MDC1 IRIF in KB2P1.21 cells, DNA damage was induced by γ-irradiation (10 Gy) 3 h prior to fixation. Subsequently, cells were washed in PBS and fixed with 4% (v/v) PFA/PBS for 20 min at room temperature (RT). Fixed cells were washed with PBS and were permeabilized for 20 min in 0.2% (v/v) Triton X-100/PBS. Subsequently, slides were washed three times with 0.2% Tween-20/PBS and blocked with staining buffer (PBS, BSA (2% w/v), glycine (0.15% w/v), Triton X-100 (0.1% v/v)) for 1 h at RT. Incubation with the primary mouse monoclonal anti-MDC1 antibody (#M2444, Sigma Aldrich) diluted 1:500 in staining buffer was carried out for 2 h in RT. Slides were then washed four times for 5 min with 0.2% (v/v) PBS-Tween-20 and then incubated with Goat anti-Mouse IgG (H + L) Cross-Absorbed Secondary Antibody, Alexa Fluor 488 (RRID: AB_2534088, #A-11029, Thermo Fisher Scientific) diluted 1:2000 in staining buffer for 1 h at RT. Slides were washed three times for 5 min with 0.2% PBS-Tween-20, once with PBS and then mounted with Duolink In Situ mounting medium with DAPI (#DUO82040, Sigma Aldrich). The same procedure was used for analysis of MDC1 and 53BP1 recruitment to irradiation-induced damage sites in RPE-1 cells. To detect the co-localization of GFP-tagged MDC1 constructs with γH2AX and 53BP1 2 h-post irradiation, slides were incubated for 1 h in RT with mouse monoclonal Anti-phospho-Histone H2AX (Ser139), clone JBW301 (#05-636, Millipore) primary antibody diluted 1:1000 in staining buffer, or with rabbit polyclonal anti-53BP1 (#ab21083, Abcam) antibody diluted 1:1000 in staining buffer. Slides were washed four times for 5 min with 0.2% PBS-Tween-20 and then incubated with Goat anti-Rabbit IgG (H + L) Cross-Adsorbed Secondary Antibody, Texas Red-X (RRID: AB_2556779, #T-6391, Thermo Fisher Scientific) diluted 1:2000 in staining buffer for 1 h at RT. Z-stack fluorescent images were acquired using the DeltaVision Elite widefield microscope (GE Healthcare Life Sciences). Multiple fields of view were imaged per sample with Olympus 100X/1.40, UPLS Apo, UIS2, 1-U2B836 or Olympus 60X/1.42, Plan Apo N, UIS2, 1-U2B933 objectives and sCMOS camera at the resolution 2048 × 2048 pixels. Deconvolution of the acquired images was performed by the softWoRx DeltaVision software. Images were analyzed and foci quantification analysis was performed using Fiji image processing package of ImageJ (1.52e). Briefly, all nuclei were detected by the “analyze particles” command and all the foci per nucleus were counted with the “find maxima” command. Data were plotted in GraphPad Prism 6 software. For the DNA damage tolerance experiment involving γH2AX detection upon treatment with mitomycin (MMC), olaparib or cisplatin, KB2P1.21 cells were seeded on glass coverslips in a 24-well plate 2 days before the treatment. To induce replication stress and DNA damage, DMSO, 300 nM MMC, 1 µM olaparib or 100 nM cisplatin were added to the medium for 24 h. Cells were then washed with PBS, fixed with 4% (v/v) PFA/PBS and further processed as described above. Z-stack fluorescent images were acquired using the DeltaVision Elite widefield microscope (GE Healthcare Life Sciences). Multiple fields of view were imaged per sample with Olympus 100X/1.40, UPLS Apo, UIS2, 1-U2B836 objective and sCMOS camera at the resolution 2048 × 2048 pixels. Deconvolution of the acquired images was performed by the softWoRx DeltaVision software. Images were analyzed and foci quantification analysis was performed as described above.

### Analysis of micronuclei formation

Cells were seeded on coverslips in 24-well plates and treated with DMSO or indicated concentrations of olaparib 24 h later. After 48 h of treatment, cells were washed with PBS and fixed with 4% (v/v) PFA/PBS for 20 min in RT. Cells were then washed 3 times in 0.2% (v/v) PBS-Tween-20 and permeabilized for 20 min in 0.2% (v/v) Triton X-100/PBS. Then, slides were washed 3 times with PBS, counterstained with DAPI (1:50000 dilution, #D1306, Life Technologies), and washed 5 times more with PBS before mounting in Fluorescence mounting medium (#S3023, Dako). Z-stack images were acquired using the DeltaVision Elite widefield microscope (GE Healthcare Life Sciences). Multiple fields of view were imaged per sample with Olympus 100X/1.40, UPLS Apo, UIS2, 1-U2B836 objective and sCMOS camera. Frequency of micronuclei positive cells was analyzed manually in Fiji.

### Total replication speed analysis using EdU incorporation

The total replication speed was assessed using the Click-iT EdU Alexa Fluor 488 Flow Cytometry Assay Kit (#C10420, Thermo Fisher Scientific) and the Click-iT EdU Cell Proliferation Kit for Imaging, Alexa Fluor 488 (#C10337, Thermo Fisher Scientific). For the quantitative flow cytometry-based analysis of EdU incorporation, KB2P1.21 cells were seeded in a 6-well plate 24 h before the experiment. The nascent DNA labeling was carried out by adding 10 µM EdU together with DMSO or 1 µM ATR inhibitor AZ20 in the medium for 30 min. Cells were then washed 3 times with PBS, collected and fixed with 4% (v/v) PFA/PBS. The next steps were carried out according to the manufacturer’s protocol. EdU intensity was measured with the BD FACSCanto II cytometer. FlowJo v10.6.1 software was used to isolate the population of single cells while excluding cell debris and cell doublets, and to export the EdU intensity values for further analysis. EdU intensity values from at least 500 cells/sample in each biological replicate were randomly selected in Excel and plotted in GraphPad Prism 6. Statistical significance was calculated using Mann-Whitney test; ****p < 0.0001. For imaging-based analysis of EdU incorporation, the Click-iT EdU Cell Proliferation Kit for Imaging, Alexa Fluor 488 was used. KB2P1.21 cells were seeded on coverslips in a 24-well plate 48 h before the experiment. The pulse labeling was performed as described above. After EdU labeling, cells were washed 3 times with PBS and fixed with 4% (v/v) PFA/PBS. The next steps were carried out according to the manufacturer’s protocol. The images were acquired using the DeltaVision Elite widefield microscope (GE Healthcare Life Sciences). Multiple fields of view were imaged per sample with Olympus 60X/1.42, Plan Apo N, UIS2, 1-U2B933 objective and sCMOS camera. The analysis of EdU intensity was performed using Fiji image processing package of ImageJ (1.52e). Briefly, all nuclei were detected by the “analyze particles” command and EdU incorporation was quantified by measuring the total signal intensity in the nuclear area. Data were plotted in GraphPad Prism 6 software. Representative cells were selected to demonstrate the differences in EdU intensities between the samples.

### Single molecule DNA fiber assay

Fork progression was measured as described previously in Schmid et al. [30] with a few modifications. Briefly, asynchronously growing sub-confluent KB2P1.21, KB1P-G3, RPE-1 or PEO1 cells were labeled with 30 μM thymidine analog 5-chloro-2’-deoxyuridine (CIdU) (#C6891, Sigma-Aldrich) for 20 min, washed three times with warm PBS and subsequently exposed to 250 μM of 5-iodo-2′-deoxyuridine (IdU) for 20 min. In the experiment assessing replication fork stability, IdU pulse was followed by adding medium containing 8 mM HU for 6 h. All cells were then collected by standard trypsinization and resuspended in cold PBS at 3.5 × 105 cells/ml. The labeled cells were mixed 1:5 with unlabeled cells resuspended in cold PBS in the concentration 2.5 × 105 cells/ml. 2.5 μl of this cell suspension were then mixed with 7.5 μL of lysis buffer (200 mM Tris-HCl, pH 7.4, 50 mM EDTA, and 0.5% (v/v) SDS) on a positively-charged microscope slide. After 9 min of incubation at RT, the slides were tilted at an ~30–45° angle to stretch the DNA fibers onto the slide. The resulting DNA spreads were air-dried, fixed in 3:1 methanol/acetic acid, and stored at 4 °C overnight. Next day, the DNA fibers were denatured by incubation in 2.5 M HCl for 1 h at RT, washed five times with PBS and blocked with 2% (w/v) BSA in 0.1% (v/v) PBST (PBS and Tween 20) for 40 min at RT while gently shaking. The newly replicated CldU and IdU tracks were stained for 2.5 h at RT using two different anti-BrdU antibodies recognizing CldU (#ab6326, Abcam) and IdU (#347580, Becton Dickinson), respectively. After washing five times with PBST (PBS and Tween 20) the slides were stained with goat the anti-mouse IgG (H + L) Cross-Adsorbed Secondary Antibody, Alexa Fluor 488 (RRID: AB_2534088, #A-11029, Thermo Fisher Scientific) diluted 1:300 in blocking buffer and with the Cy3 AffiniPure F(ab’)₂ Fragment Donkey Anti-Rat IgG (H + L) antibody (#712-165-513, Jackson ImmunoResearch) diluted 1:150 in blocking buffer. Incubation with secondary antibodies was carried out for 1 h at RT in the dark. The slides were washed five times for 3 min in PBST, air-dried and mounted in Fluorescence mounting medium (#S3023, Dako). Multiple fields of view from at least two slides (technical replicates) of each sample were imaged using the Olympus 60X/1.42, Plan Apo N, UIS2, 1-U2B933 objective and sCMOS camera at the resolution 2048 × 2048 pixels. Replication fork stability was analyzed by measuring the track lengths of CldU and IdU separately and by calculating IdU/CldU ratio. In the replication fork restart experiment stalling with 2 mM hydroxyurea (HU) was performed for 60 min between the CldU and IdU pulse labels. After three washes with warm PBS, IdU pulse was carried out for 40 or 80 min. Samples were then processed as described above. Replication fork restart efficiency was then analyzed by manual counting of CldU tracks only (stalled forks) and CldU + IdU tracks (restarted forks) in ImageJ.

### Proximity ligation assay (PLA)

KB2P1.21 cells were seeded on sterile glass coverslips in a 24-well plate 48 h before the experiment. EdU labeling was performed by adding 10 μM EdU in the medium for 30 min. Cells were then washed three times with PBS, pre-extracted for 5 min with CSK buffer (25 mM HEPES, pH 7.5, 50 mM NaCl, 1 mM EDTA, 3 mM MgCl_2_, 300 mM sucrose and 0.5% (v/v) Triton X-100) on ice and fixed with 4% (v/v) PFA/PBS for 15 min at RT. After three washes with PBS, permeabilization was performed with 100% ice cold methanol for 20 min at -20 °C. Slides were then washed three times with PBS and EdU was detected according to the manufacturer’s protocol (Click-iT EdU Cell Proliferation Kit for Imaging, Alexa Fluor 488, #C10337, Thermo Fisher Scientific). Next, slides were blocked with staining buffer (PBS, BSA (2% w/v), glycine (0.15% w/v), Triton X-100 (0.1% v/v)) for 1 h at RT. Incubation with the primary antibodies mouse monoclonal anti-MDC1 antibody (#M2444, Sigma Aldrich) diluted 1:500 in staining buffer and rabbit monoclonal anti-PCNA (D3H8P) XP antibody (#13110, Cell Signaling Technology) diluted 1:800 in staining buffer was carried out at 4 °C overnight. The next day, the slides were washed four times with 0.2% (v/v) Tween-20/PBS for 5 min and in situ proximity ligation was performed according to the Duolink Detection Kit protocol (#DUO92101, Sigma Aldrich). Z-stack images were acquired using the DeltaVision Elite widefield microscope (GE Healthcare Life Sciences). Multiple fields of view were imaged per sample with Olympus 100X/1.40, UPLS Apo, UIS2, 1-U2B836 objective and sCMOS camera. All nuclei were detected by the “analyze particles” command, only the EdU positive nuclei were then selected for further analysis. The PLA foci were then quantified with the “find maxima” command. Data were plotted in GraphPad Prism 6. For siRNA-mediated knockdown cells were seeded after 48 h post-transfection and experiment performed at 72 h post-transfection.

### In situ analysis of protein interactions at DNA replication forks (SIRF)

RPE-1 cells were seeded on sterile glass coverslips in a 24-well plate 48 h before the experiment in the density of 30 000 cells/well. EdU labeling was performed by adding 25 μM EdU in the medium for 10 min. After three washes with PBS, replication stress was induced in the HU-treated samples by adding 2 mM HU for 2 h. Cells were then washed twice with PBS and nuclei were pre-extracted with CSK buffer (10 mM PIPES pH 7, 0.1 M NaCl, 0.3 M sucrose, 3 mM MgCl_2_, 0.5% (v/v) Triton X-100) on ice for 5 min. After pre-extraction, cells were washed with PBS and fixed in 4% (v/v) PFA/PBS for 15 min at RT. After three washes with PBS, permeabilization was performed with 0.2% (v/v) Triton X-100 for 15 min at RT. Then, click reaction was performed by adding the click reaction buffer (100 mM Tris pH 8, 4 mM CuSO_4_, 100 mM sodium ascorbate, 50 µM biotin-azide) to the samples and incubating at 37 °C for 2 h. Slides were then incubated in the blocking buffer (PBS, BSA (2% w/v), glycine (0.15% w/v), Triton X-100 (0.1% v/v)) for 1 h at 37 °C, followed by incubation with primary antibodies mouse anti-MDC1 (#M2444, Sigma Aldrich, diluted 1:200) and rabbit anti-biotin (D5A7, #5597, Cell Signaling, diluted 1:1000) overnight at 4 °C. For MRE11 SIRF foci, primary antibodies rabbit anti-MRE11 (NB100-142, Novus, diluted 1:250) and mouse anti-biotin (200-002-211, Jackson IR, diluted 1/200) were prepared for overnight incubation at 4 °C. The next steps are identical to the steps described in the PLA protocol above.

### Immunoblotting

200.000 RPE-1 cells were seeded in a 6-well plate 24 h prior the experiment. Replication stalling was induced by adding 1 mM hydroxyurea (HU) into the medium for 24 h. Cells were then washed with PBS, trypsinized and collected in the 15 ml tubes. After washing with PBS, cells were lysed for 40 min in RIPA buffer supplemented with Halt Protease and Phosphatase Inhibitor Cocktail (100x) (#78420, Thermo Fisher Scientific) while briefly vortexed every 10 min. Lysates were then centrifuged at 10.000 rpm for 10 min at 4 °C and the supernatant was collected to determine protein concentration using Pierce BCA Protein Assay Kit (#23225, Thermo Fisher Scientific). Before loading, protein lysates were denatured at 95 °C for 5 min in 6x SDS sample buffer. Proteins were separated by SDS/PAGE in 10% gel before wet transfer to 0.45 µm nitrocellulose membranes (GE Healthcare) and blocked in 5% dry milk powder in TBS-T (100 mM Tris, pH 7.5, 0.9% NaCl, 0.05% Tween-20). Membranes were incubated with the following primary antibodies: primary mouse monoclonal anti-MDC1 antibody (1:1000 #M2444, Sigma Aldrich), Vinculin (E1E9V) XP® Rabbit mAb (1:1000, #13901, Cell Signaling), rabbit polyclonal anti-RPA32 (1:1000, #A300-244A, Bethyl Laboratories), rabbit recombinant monoclonal anti-phospho RPA32 (S4/S8) (1:1000, #A700-009, Bethyl Laboratories), rabbit polyclonal anti-CHK1 (1:5000, #NB100-464, Novus Biologicals), rabbit monoclonal anti-phospho CHK1 (S345) (133D3) (1:1000, #2348, Cell Signaling), rabbit polyclonal anti-KAP1 (1:1000, #NB500-158, Novus Biologicals), rabbit polyclonal anti-phospho KAP1 (S824) (1:1000, #A300-767A, Bethyl Laboratories), rabbit anti-MRE11 (1:5000, NB100-142, Novus), mouse anti-SMARCAL1 (1:1500, sc-376377, Santa Cruz), rabbit anti-RECQ1 (1:1000, A300-447A-T, Bethyl), rabbit polyclonal anti-γ-Tubulin (1:1000, #5886, Cell Signaling) and mouse monoclonal anti-PARP1 (1:500, #MA3-950, Thermo Fisher Scientific). The antibodies were diluted in 5% milk in TBS-T and incubated with membranes for 2 h at RT. After three 5 min washes in TBS-T, anti-mouse or anti-rabbit Horseradish Peroxidase (*HRP*)-linked secondary antibodies (Cell Signaling, dilution 1: 2500) were applied for 1 h at room temperature. Images were acquired using Vilber FUSION FX chemiluminescent imager.

### Transmission electron microscopy of replication intermediates (RI)

The procedure was performed as described previously [31] with minor modifications. A total of 2.5–5.0 × 106 asynchronously growing sub-confluent RPE-1 cells were harvested by trypsinization and resuspended in 10 mL of cold PBS. DNA was cross-linked by exposing the living cells twice to 4,5′,8-trimethylpsoralen at a final concentration of 10 μg/mL followed by 3 min irradiation pulses with UV 365-nm monochromatic light (UV Stratalinker 1800, Agilent Technologies). The cells were then washed repeatedly with cold PBS and lysed with a cell lysis buffer (1.28 M sucrose, 40 mM Tris-Cl, pH 7.5, 20 mM MgCl2, and 4% (v/v) Triton X-100). The nuclei were then digested in a digestion buffer (800 mM guanidine-HCl, 30 mM Tris-HCl, pH 8.0, 30 mM EDTA, pH 8.0, 5% (v/v) Tween 20, and 0.5% (v/v) Triton X-100) supplemented with 1 mg/mL proteinase K at 50 °C for 2 h. Genomic DNA was extracted with a 24:1 Chloroform:Isoamyl alcohol mixture by phase separation (centrifugation at 8.000 rpm for 20 min at 4 °C) and precipitated by addition of equal amount of isopropanol to the aqueous phase, followed by another centrifugation step (8.000 rpm for 10 min at 4 °C). The obtained DNA pellet was washed once with 1 mL of 70% ethanol, air-dried at RT, and resuspended by overnight incubation in 200 μL TE (Tris-EDTA) buffer at RT. 12 μg of the extracted genomic DNA was digested for 5 h at 37 °C with 100 U restriction enzyme PvuII high-fidelity (#R3151S, New England Biolabs). The digest was cleaned up using a silica bead DNA gel extraction kit (#K0513, Thermo Fisher Scientific). The benzyldimethylalkylammonium chloride (BAC) method was used for native spreading of the DNA on a water surface and then loading it on carbon-coated 400-mesh magnetic nickel grids. After the spreading procedure, the electron density of the DNA was increased by platinum coating with the platinum-carbon rotary shadowing technique using the MED 020 High Vacuum Evaporator (Bal-Tec). The grids were then scanned in a semi-automated fashion using a transmission electron microscope (FEI Thalos 120, LaB6 filament) at high tension ≤120 kV and pictures were acquired with a bottom mounted CMOS camera BM-Ceta (4000 × 4000 pixels). The images were processed with MAPS Version 3.14 (Thermo Fisher) and analyzed using MAPS Offline Viewer Version 3.14.11 (Thermo Fisher). Mean + SD values of analyzed frequency of RIs obtained from three independent replicates were plotted and statistics was performed in GraphPad Prism 9 using unpaired t test.

### Quantification and statistical analysis

Statistical parameters including sample size, number of biological replicates, applied statistical tests and statistical significance are reported in the figures, corresponding figure legends and Method Details sections.

### CRISPR/Cas9 genetic screens

Sequence alignment and enrichment analysis done by comparing day 0 vs PARPi-treated population from six replicates (independent mutagenesis) was carried out using MAGeCK software [26].

### Clonogenic assays

See Figs. 1B, C, E, 4D, E, S1C, S1D, S1H, S4G and S5G. All experiments indicated in these figures were performed as three replicates and graphs were drawn from these data using GraphPad prism 6. Cell survival in each condition was normalized to the corresponding DMSO-treated control. Statistical analysis was performed in Graphpad Prism using a Two-way ANOVA followed by Dunnet’s test; *p < 0.05, **p < 0.01, ***p < 0.001; ****p < 0.0001. Analysis of the competition assays in Fig. S1B, F was carried out by comparing the frequency of modifications in gRNA1/2-targeted sites in DNA samples from *Mdc1*-targeting gRNA1/2 cells before and after PARPi treatment, and NT gRNA cells collected at day 0 of the experiment.

### CTB proliferation assay

See Fig. S4C, all the measured values were obtained from at least three independent biological replicates. Fluorescence measured in each condition was normalized to the background fluorescence of CellTiter Blue reagent incubated in an empty well with growth medium. The data were plotted using GraphPad Prism 6.

### In vivo studies

See Figs. 1F and S2F. For the in vivo transplantation experiments, mice were stratified into the different treatment arms by random allocation into the vehicle or PARPi treated group. 10 mice were used in each group (Non-targeting gRNA or *Mdc1*-targeting gRNA1; vehicle- or olaparib-treated) in Fig. 1F. 5 mice were used in each of the four group (Non-targeting gRNA or *Mdc1*-targeting gRNA1; vehicle- or AZD2461-treated) in Fig. S2F. Kaplan Meyer survival curves were plotted and statistical analysis was performed using log-rank test in GraphPad prism 6. For the in vivo experiments, treatment of mice with the modified tumors was performed blind. Blinding was not applied to any other experiment. Unbiased results were obtained from the combination of multiple repetitions from independent researchers.

### Immunofluorescence

**Fig. 2:**
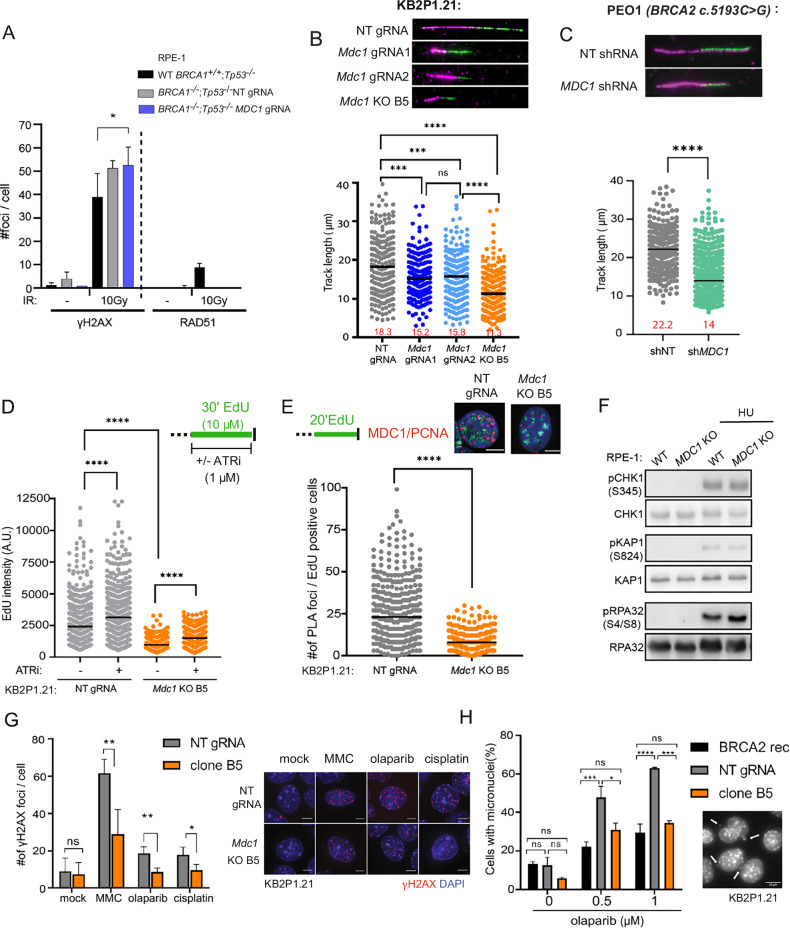
MDC1 deficiency reduces replication fork speed and improves the DNA damage tolerance of BRCA2-deficient cells. **A** Quantification of RAD51 foci formation in RPE-1 cells 4 h upon irradiation with 10 Gy. Mean + SD of three independent experiments is shown. Two-way ANOVA followed by Tukey’s multiple comparison test; *p < 0.05. **B** DNA fiber assay in KB2P1.21 cells with representative images of individual fibers. Median of track lengths is shown. Similar results were obtained from three independent experiments. Mann-Whitney test; ns = non-significant, ***p < 0.001, ****p < 0.0001. **C** DNA fiber assay in PEO1 cells with representative images of individual fibers. Median of track lengths is shown. Similar results were obtained from three independent experiments. Mann-Whitney test; ****p < 0.0001. **D** The total replication speed assay. Median of EdU intensity measured in 500 KB2P1.21 cells using a cytometer is shown. Similar results were obtained from three independent experiments. Mann-Whitney test; ****p < 0.0001. **E** Representative images and analysis of proximity ligation assay showing the interaction of MDC1 with PCNA (red) in EdU-positive cells (green). Median of values from three independent experiments is shown. Scale bars represent 5 µm. Mann-Whitney test; ****p < 0.0001. **F** Western blotting of DDR markers before and after 1 mM HU for 24 h in WT and *MDC1* knockout RPE-1 cells. Similar results were observed in three independent experiments. **G** Quantification and representative images of γH2AX foci in the KB2P1.21 cells after 24 h treatment with DMSO, 300 nM mitomycin C (MMC), 1 µM olaparib or 100 nM cisplatin. Mean ± SD of four independent experiments is shown. The scale bar represents 10 µm. Two-way ANOVA followed by Dunnet’s multiple comparison test; ns= non-significant, *p < 0.05, **p < 0.01. **H** Percentage of micronuclei positive KB2P1.21 cells after 48 h treatment with DMSO or olaparib. Mean ± SD of values from three independent experiments is shown. A representative image with examples of micronuclei; the scale bar represents 10 µm. Two-way ANOVA test; *p < 0.05, **p < 0.01, ***p < 0.001, ****p < 0.0001.

See Figs. 2A, E, G, H, 4A, S1A, S3E, F, G, S4A, D. Each condition was stained as indicated in the method details section. GE DeltaVision fluorescent microscope with 60× or 100× objectives with immersion oil were used to acquire images. Each image was taken in at least 6 Z-layers and maximum intensity Z-projection was performed in Fiji to create a single layer for quantification or for preparation of representative images. Foci were quantified in the Figs. 2A, E, G, S1A, S3E, and S4D using the following procedure: Nuclei were segmented by manual intensity thresholding in the DAPI channel and separation using the “watershed” function. Detection of individual nuclei for analyzes was performed by “Analyze particles” function. Number of foci per cell was quantified by “find maxima” function in each defined particle. A minimum of 100 cells were quantified from each condition and the average number of foci were plotted in GraphPad Prism 6. Statistical analysis in Figs. 2E, S3E, and S4D was performed using Mann-Whitney test; ****p < 0.0001. Analyzes in Fig. 2A was performed using Two-way ANOVA followed by Tukey’s multiple comparison test; *p < 0.05. Analyzes in Fig. 2G was performed using Two-way ANOVA followed by Dunnet’s multiple comparison test; ns = non-significant, *p < 0.05, **p < 0.01. In Fig. 2H, micronuclei positive cells were counted manually, and statistical significance was calculated using the Two-way ANOVA test; *p < 0.05, **p < 0.01, ***p < 0.001; ****p < 0.0001.

### Single molecule DNA fiber assay

**Fig. 3:**
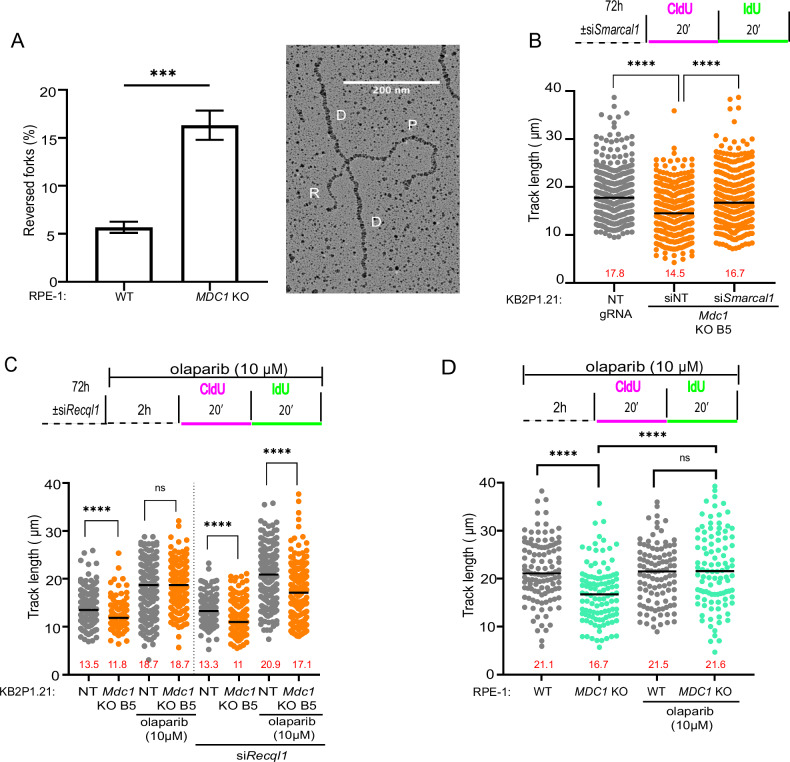
Reduced fork speed in MDC1 deficient cells is associated with changes in fork reversal. **A** Frequency of RVFs in untreated WT or *MDC1* knockout RPE-1 cells analyzed by transmission electron microscopy. Mean ± SD of three independent experiments is shown. An electron micrograph of a representative RVF. (D: daughter strand; P: parental strand; R: regressed arm) Unpaired t-test; ***p < 0.001. **B** Total track length in KB2P1.21 cells without and after *Smarcal1*-targeting siRNA treatment. Median of three independent experiments is shown. Mann-Whitney test; ****p < 0.0001. **C** Total track length in KB2P1.21 cells targeted with NT or *Recql*-targeting siRNA after treating with DMSO or 10 µM olaparib. Median of three independent experiments is shown. Mann-Whitney test; ns= non-significant, ****p < 0.0001. **D** Track length analysis in RPE-1 cells after treatment with DMSO or 10 µM olaparib. Median of at least 100 fork lengths is shown. Similar results were obtained in three independent experiments. Mann-Whitney test; ns= non-significant, ****p < 0.0001.

**Fig. 4:**
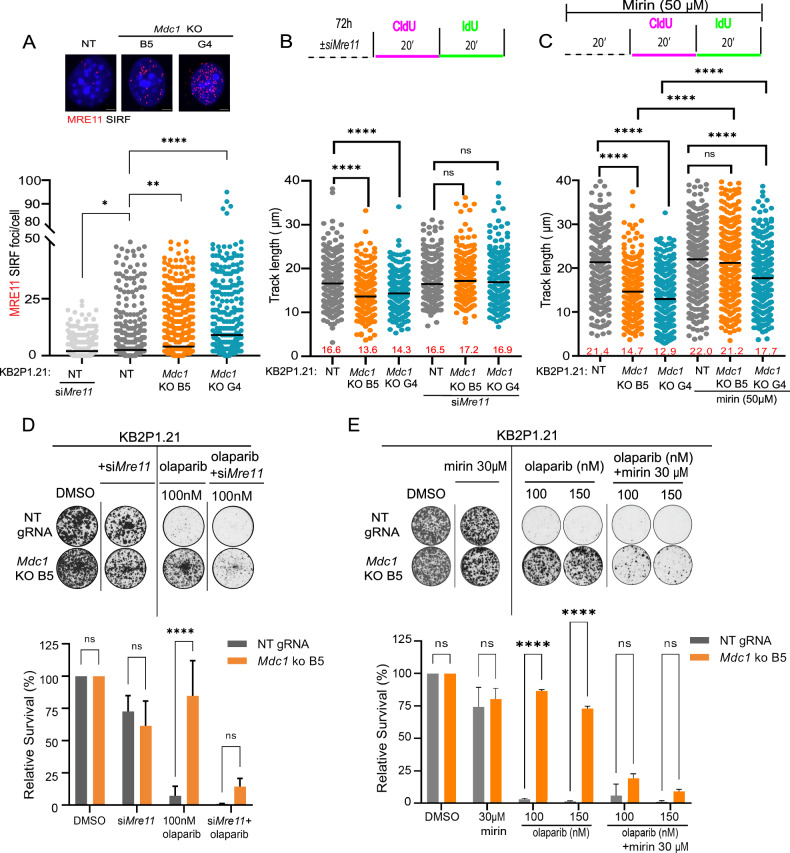
MDC1 regulates replication fork speed and PARPi sensitivity by suppressing MRE11 activity. **A** Analysis of MRE11 localization at active replication forks by in situ analysis of protein interactions at DNA replication forks (SIRF). Representative images and quantification are shown. Similar results were obtained from at least three independent experiments. The scale bars represent 2 µm. One-way Anova test; *p < 0.05, **p < 0.01, ****p < 0.0001. **B** Total track length in KB2P1.21 cells without and after si*Mre11* treatment. Median of three independent experiments is shown. Mann-Whitney test; ns= non-significant, ****p < 0.0001. **C** Total track length in KB2P1.21 cells without and after treatment with 50 µM mirin. Median of three independent experiments is shown. Mann-Whitney test; ns= non-significant, ****p < 0.0001. **D** Representative images and quantification of clonogenic assay with siRNA-mediated MRE11 knockdown on KB2P1.21 cells after treatment with olaparib. Mean ± SD of three independent experiments is shown. Two-way ANOVA test; ns= non-significant, ****p < 0.0001. **E** Representative images and quantification of clonogenic assay with KB2P1.21 cells showing treatment response to mirin or olaparib alone, and to a combined treatment. Mean ± SD of three independent experiments is shown. Two-way ANOVA test; ns= non-significant, ****p < 0.0001.

See Figs. 2B, C, 3B, D, E, 4B, C, S3A–D, S5C–F. The nascent DNA pulse labeling, fiber spreading and processing of slides was performed as described in the “Method” section. To assess fork progression CldU + IdU track lengths of ~80 fibers per sample were measured using the line tool in ImageJ software. Statistical analysis was carried out using GraphPad Prism 6 using Mann-Whitney test; *p < 0.05, **p < 0.01, ***p < 0.001; ****p < 0.0001.

### Immunoblotting

See Figs. 2F, 3C, S1G, S2C, S4E, and S5D. Immunoblotting experiments were performed at least twice. Representative images are shown.

## Results

### *Mdc1* depletion causes PARPi resistance in vitro and in vivo

To identify mechanisms of PARPi resistance in KB2P tumors, we carried out a functional genetic screen in the KB2P3.4 cell line, which is derived from a treatment-naïve *Brca2*^*-/-*^*;Trp53*^*-/-*^ mouse mammary tumor [19]. In these cells we introduced the pool B of the mouse GeCKO v2 library, a CRISPR/Cas9-based single plasmid system (lentiCRISPRv2) targeting 20,628 mouse genes [24]. Transduced cells were then selected for 3 weeks with 200 nM of the PARPi AZD2461 (Fig. 1A), a concentration that kills at least 90% of the parental cells (data not shown). The advantage of AZD2461 is that it is a poor substrate for P-glycoprotein (P-gp), hence cells with increased P-gp expression are not positively selected throughout the screen [32]. Sequencing of the PARPi-surviving populations of 6 biological replicates of this screen revealed a reproducible enrichment of sgRNAs targeting *Mdc1*. The strong effect of *Mdc1* depletion is shown by its high RRA score of 5.78 × 10^−7^ among all positively selected genes, as determined by the MAGeCK algorithm [26] (Fig. 1A; Table S1).

To validate the role of MDC1 in PARPi resistance in BRCA2-defective cells, we used an independent *Brca2*^*-/-*^*;Trp53*^*-/-*^ mouse mammary tumor cell line (KB2P1.21) to generate polyclonal KB2P1.21 cell lines by targeting the *Mdc1* gene with two different gRNAs. We then determined the targeting efficacy by TIDE (Tracking of Indels by Decomposition) analysis [28] and immunofluorescence staining of MDC1 foci formation upon irradiation (Fig. S1A). Both gRNAs yielded a frame-shift modification rate above 50% leading to a significant reduction of MDC1 foci (Fig. S1A, B). We also successfully isolated two homogeneous knockout (KO) cell lines (hereafter called B5 and G4) with a complete abrogation of MDC1 foci (Fig. S2A). The MDC1-deficient lines showed a significantly improved survival in response to PARPi exposure, with the clonal lines displaying the strongest resistance phenotype (Fig. 1B, C). These results were corroborated by a significant increase in the frequency of alleles with *Mdc1* frameshift modifications following PARPi treatment in the polyclonal cells (Fig. S1B). We also confirmed the PARPi resistance phenotype in MDC1-deficient derivatives of the KB2P3.4 cell line used for the CRISPR/Cas9 screen (Figs. S1D, E and S1F). Of note, the observed PARPi resistance is independent of alterations in PARP1 expression (Fig. S1G). Importantly, MDC1 deficiency does not confer resistance specifically to PARPi, as we also observed a significantly reduced sensitivity to cisplatin (Fig. S1C). Interestingly, MDC1 deficiency also causes PARPi resistance in the context of BRCA1 deficiency as demonstrated using the human retinal pigment epithelium RPE-1 cells and mouse mammary tumor-derived KB1P-G3 cells (Figs. 1D, E; S1H, I and S2B). Moreover, shRNA-mediated depletion of *MDC1* resulted in olaparib resistance in the human high-grade serous ovarian cancer cell line PEO1, that carries the *BRCA2 c.5193C*>*G* mutation leading to a complete loss of protein stability (Fig. 1F, G). To further investigate whether the PARPi resistance can be recapitulated in vivo, we expressed the non-targeting (NT) or *Mdc1*-targeting gRNAs in the KB2P tumor-derived organoids and grafted them orthotopically into a mammary gland of mice as described previously [23]. Even though the *Mdc1*-targeting efficiency in the organoids was incomplete (43% and 40% respectively, Fig. S2D, E), we observed a significantly reduced survival of animals bearing the *Mdc1*-targeted KB2P organoid-derived tumors in response to both olaparib and AZD2461 (Figs. 1H and S3F). Together, these findings show that loss of MDC1 counteracts the efficacy of PARPi in BRCA1/2-deficient tumor cells both in vitro and in vivo.

### MDC1 deficiency reduces replication fork velocity and improves DNA damage tolerance in BRCA2-deficient cells

We and others have previously shown that restoration of HR is a frequent mechanism of chemoresistance [8, 15–18]. Importantly, MDC1; BRCA1; p53-deficient RPE-1 cells did not regain the ability to form RAD51 ionizing radiation-induced foci (IRIF) (Fig. 2A). Another proven mechanism of PARPi resistance in BRCA2-deficient cells is the restoration of RF stability [10, 11]. Interestingly, MDC1-deficient KB2P1.21 cells did not exhibit restored fork protection following HU-induced fork stalling in contrast to our previous finding in H2AX-deficient KB2P cells (Fig. S3A) [11]. Instead, we noticed a marked decrease in RF progression, even in unperturbed conditions (Figs. 2B and S3B). We also observed a significant level of RF speed reduction in BRCA1-deficient KB1P-G3 cells expressing *Mdc1*-targeting gRNA (Fig. S3C). Additionally, the *MDC1-*depleted PEO1 cells showed slower fork speed confirming the phenotype in a *BRCA-*mutated human cancer line (Fig. 2C). A similar reduction in RF velocity has also been reported in normally growing, BRCA1/2-proficient cells that lost the DNA damage response proteins RNF8, RNF168, and 53BP1 [30]. When we tested the effects of MDC1 loss in BRCA1/2-proficient human RPE-1 cells, we also observed a significant reduction in RF progression (Fig. S3D, E). This strongly suggests that the function of MDC1 in controlling RF speed is BRCA-independent. To identify which MDC1 domain is important to control replication fork progression, we introduced various *MDC1* functional domain mutants into *MDC1* KO RPE-1 cells [21] (Fig. S3D, F, G). While RF velocity was fully rescued by wild-type MDC1 as well as FHA, SDTD, TQXF, and PST mutants, this was not the case for the MDC1 mutant lacking the C-terminal BRCT domains responsible for chromatin binding (Fig. S3D). These data suggest that the BRCT domains of MDC1 are essential for maintaining normal RF progression.

MDC1, together with its interacting partner TOPBP1, has previously been shown to play a role in the activation of the intra-S phase checkpoint upon induction of replication stress [33, 34] To investigate whether the reduced RF velocity observed upon loss of MDC1 is a consequence of unscheduled firing of late/dormant origins of replication due to an impaired intra S-phase checkpoint, we assessed the total rate of EdU incorporation in intact nuclei from KB2P1.21 cells (Figs. 2D and S4A). The changes in the rates of DNA synthesis at individual RFs and the total DNA synthesis have been previously shown to be inversely correlated upon release of new origins [35, 36]. Indeed, a significant increase in the EdU intensity was observed in both wild-type (WT) and *Mdc1* KO cells by ATR inhibition, which is known to unleash massive origin firing [37, 38]. Compared to WT cells, the *Mdc1* KO cells showed a strong reduction in total EdU intensity in both untreated and ATRi-treated conditions (Fig. 2D). Moreover, we also directly measured the inter-origin distance (IOD) by single-molecule analysis of replication tracts. No clear difference in IOD was observed in *Mdc1* KO cells (Fig. S4B). Collectively, these results demonstrate that changes in origin firing cannot explain the observed reduction in RF speed. Importantly, the slower replication rate in the *Mdc1* KO KB2P1.21 cells does not correlate with a significant change in the cell proliferation rate (Fig. S4C).

Since our results suggest that the effect of MDC1 loss on RF dynamics is independent of the activation of the intra-S phase checkpoint, we performed proximity ligation assays (PLA) and in situ analysis of protein interactions at DNA replication forks (SIRF) to determine whether MDC1 regulates RF dynamics by physically interacting with the replication machinery. SIRF is a proximity ligation-based technique used to monitor the physical interaction between the nascent DNA (EdU-labeled) and the protein of interest at single-cell resolution. Similar to PLA, we can quantify the fluorescently labeled products. Consistent with a direct role for MDC1 in the regulation of RF progression, we detected SIRF foci in MDC1-proficient compared to MDC1-deficient KB2P1.21 and RPE-1 cells indicating that MDC1 directly localizes to active RFs during unchallenged DNA replication (Figs. 2E and S4D). Interestingly, MDC1 localization at RFs increases following replication stress induced by 2 mM HU (Fig. S4D), suggesting different modes of action for MDC1 during normal and stressful DNA replication.

To investigate the impact of fork slowing in MDC1-deficient cells on global DNA damage response activation, we investigated typical hallmarks of DNA damage and replication stress, such as phosphorylation of RPA, KAP1 and CHK1 in WT and *MDC1* KO RPE-1 cells. Consistent with previous results obtained in RNF168-deficient cells [30], slower RF progression during normal S phase in *Mdc1*-depleted cells is not associated with replication stress and global DDR activation (Fig. 2F). Similar results were obtained in *Mdc1* KO BRCA2; p53-deficient KB2P1.21 cells (Fig. S4E). Interestingly, 24 h treatment of MDC1-deficient KB2P1.21 cells with the replication-targeting agents mitomycin C (MMC), olaparib or cisplatin caused an ~50% reduction in the number of the γH2AX foci, relative to MDC1-proficient KB2P1.21 cells (Fig. 2G). This cannot be explained by a reduced ability of cells to form γH2AX foci in the absence of MDC1 (Figs. 2A and S3E). Compared to MDC1-proficient KB2P1.21 cells, MDC1-deficient cells also showed significantly fewer cells with micronuclei formation following 48 h exposure to olaparib (Fig. 2H). These data show that slower RF progression in BRCA2-deficient cells with MDC1 loss does not impose a threat to genome integrity in unperturbed conditions and rather improves DNA damage tolerance.

### Loss of MDC1 leads to an accumulation of reversed forks and delay in restart

We next sought to investigate the genetic basis of replication fork slowing in MDC1-deficient cells. Prior literature has established a strong association between reduced fork velocity and the formation or stabilization of reversed replication forks (RVFs) [31, 39]. Fork reversal is a highly dynamic process enabling cells to attenuate the cytotoxic effects of a wide range of replication stress-inducing stimuli of endogenous and exogenous origin [13, 40]. Alterations in the remodeling or resolution of RVF, particularly changes affecting the protection of regressed arms, can strongly influence fork restart once the replication stress is alleviated [12, 41–44]. To investigate whether reduced RF speed in MDC1-deficient cells is accompanied by an accumulation and stabilization of RVF intermediates, we directly quantified RFs during normal replication using electron microscopy (EM). Consistent with our hypothesis, while we detected only 5% of the RVF intermediates in WT RPE-1 cells, an ~3-fold higher frequency was observed in the *MDC1* KO cells (Fig. 3A). A similar level of RVF frequency upon loss of other DDR factors has previously been reported [30]. Spontaneous fork reversal was also accompanied by increased fork asymmetry in MDC1-deficient cells (Fig. S4F), possibly due to the instability of reversed forks in the context of BRCA deficiency [10, 11].

To pinpoint the molecular players driving this fork reversal phenotype in MDC1-deficient cells, we depleted SMARCAL1, a key fork remodeler known to catalyze regression of stalled forks and preserve genome integrity [45, 46]. Knockdown of *Smarcal1* indeed rescued fork speed and PARPi sensitivity in MDC1-deficient cells (Figs. 3B; S4G, H), establishing a direct mechanistic link between SMARCAL1-dependent fork reversal and the slowed fork progression/PARPi resistance phenotype observed upon MDC1 loss.

Fork reversal and restart are opposing processes that are tightly balanced by distinct enzymatic activities [47]. PARP1 plays a pivotal role in this balance by stabilizing reversed forks through transient inhibition of the RECQ1 helicase PARP1 plays a pivotal role in this balance by stabilizing RVFs in the regressed state through transient inhibition of the RECQ1 helicase [12]. Using a high PARPi concentration of 10 μM olaparib – a 100-fold higher concentration than used in our clonogenic survival assays (Figs. 1B–G; S1D, H)—this inhibitory effect of PARP1 on RECQ1-mediated RF restart can bypass the reversed state [12]. Indeed, treatment with 10 μM olaparib rescued fork speed defects in both MDC1-deficient mouse KB2P1.21 cells and in human RPE-1 cells and (Fig. 3C, D). To verify whether the rescue effect obtained by PARPi treatment was due to the transient inhibition of RECQ1, we knocked down *Recq1* using siRNA. Indeed, *Mdc1* KO cells showed slower fork speed even after high concentration of olaparib treatment in RECQ1-depleted cells (Fig. 3C), confirming that the PARPi-mediated acceleration of fork speed is driven by fast RECQ1-mediated branch migration. Collectively, these data demonstrate that MDC1 loss increases spontaneous SMARCAL1-mediated fork reversal, and that RECQ1-driven restart dynamics further modulate replication speed in this background.

Additionally, we tested whether the increased RVF frequency might be explained by altered dynamics in the restart of replication forks following transient fork stalling (Fig. S5A). Consistently, a transient restart delay was detected in the MDC1-deficient KB2P1.21 B5 and G4 cell lines at 40 min recovery after HU removal (Fig. S5B). Together, these results show that MDC1 is important for the regulation of RF restart upon replication stress-induced fork reversal.

### MDC1 deficiency reduces replication fork speed and promotes PARPi resistance by regulating MRE11 activity

In previous studies, the nuclease MRE11 was shown to act at replication forks during unperturbed S phase affecting the rate of fork progression [30, 41]. We therefore hypothesized that MDC1 may antagonize MRE11-mediated fork processing leading to reversed fork stabilization and active fork slowing. Given the essential role of MDC1 in recruiting the MRN complex near DSB (through its SDTD motif) [48], and given that the MDC1 mutant lacking the SDTD motif could still rescue fork slowing in MDC1 KO cells (Fig. S3D), we hypothesized that, during unperturbed S phase, MRE11 action might not require MDC1 for its localization near RFs. To test this hypothesis, we quantified MRE11 localization at forks in the absence of MDC1 by SIRF assay. Consistent with our hypothesis, SIRF analysis showed that MRE11 localization is increased at stalled forks in *Mdc1* KO cells during unchallenged DNA replication (Fig. 4A). This result indicates that, in contrast to DSB, MRE11 localizes at replication forks in an MDC1-independent manner.

To test whether MRE11-mediated processing is required for fork slowing, we silenced MRE11 in KB2P1.21 using siRNA. Indeed, MRE11 abrogation resulted in full restoration of fork speed in MDC1-deficient cells (Fig. 4B). Similar results were recapitulated using the MRE11 selective inhibitor mirin (Fig. 4C). We previously showed that loss of fork reversal by DNA-PK inhibition can sensitize MDC1-deficient cells to the PARPi olaparib [49]. We then asked whether MRE11 inhibition can exhibit a similar effect and re-sensitize MDC1-deficient tumors to PARPi. While continuous MRE11 inactivation as monotherapy did not have a clear effect on the survival of KB2P1.21 cells regardless of *Mdc1* status, we observed sensitization of MDC1-deficient cells to olaparib when combined with MRE11 knockdown or mirin (Fig. 4D, E). Hence, PARPi resistance caused by reduced replication speed and subsequent DNA damage tolerance of *Mdc1*-depleted cells can be counteracted by blocking MRE11.

To gain more insight into the underlying mechanism of the delayed recovery of stalled RFs, we measured the track length of the restarted forks after HU treatment. As expected, significantly shorter IdU tracks were measured in the native and HU-treated recovery conditions in both MDC1-deficient KB2P1.21 lines B5 and G4. We also limited MRE11 nuclease activity and measured IdU track lengths using the same experimental settings. Both siRNA-mediated *Mre11* depletion and mirin-induced MRE11 inhibition resulted in longer track length in MDC1-deficient cells (Fig. S5C, D). The effect of MRE11 inhibition on fork progression was also quantified in RPE-1 cells. Indeed, the rescue of fork speed could be confirmed in these human cells after mirin treatment (Fig. S5E).

Previous literature has shown that MRE11 acts in concert with the nucleases EXO1 and DNA2 in the degradation of RVF [42, 50]. We then decided to study the involvement of other nucleases in the RF slowing in MDC1-deficient cells. While treatment with the selective DNA2 inhibitor (C5) [51] failed to restore normal RF speed, treatment with the EXO1 inhibitor LNT1 [52] completely rescued fork speed (Fig.S5F) and PARPi sensitivity in MDC1-deficient cells (Fig. S6A). Since LNT1 also inhibits FEN1 [52], we confirmed the specific role of EXO1 on fork speed and PARPi response by genetic depletion with siRNA (Fig. S6B, C). These data indicate that MDC1 restrains MRE11 and EXO1 nuclease activity, whose coordinated action is required for downstream replication fork reversal and slowdown of replication speed.

## Discussion

In this study, we identified loss of the DSB repair protein MDC1 as a novel mechanism leading to PARPi resistance in BRCA1/2-deficient cells in vitro and in BRCA2-deficient tumors in vivo, consistent with a recent report [53]. Thus far, the function of MDC1 in RF metabolism was unknown while its role in canonical DSB repair is well documented [54, 55]. Using iPOND assays, previous reports showed that MDC1 to be present at RFs in response to replication fork stalling [56, 57].

Here, we show that MDC1 is already present at RFs during unperturbed S phase, where it contributes to RF progression. Reduction in fork speed and regressed fork accumulation observed in MDC1-deficient cells highlight the importance of this protein in RF biology. Mechanistically, MDC1 dampens MRE11-mediated processing to modulate fork speed. In response to DNA replication stress, MRE11 has been traditionally linked to the degradation of reversed RFs [42, 46]. Therefore, the increased MRE11 recruitment to active forks along with the stabilization of reversed replication fork intermediates was largely unexpected. Together with recent reports [43, 58], our results showing that MRE11 loss rescues replication fork speed in MDC1-deficient cells provide direct evidence that MRE11 may function in different steps of fork reversal. Specifically, MRE11 might degrade pre-existing nicks/gaps leading to the formation of ssDNA required for further strand annealing and SMARCAL1-mediated fork reversal. Once fork remodelers engage and stalled forks are actively remodeled, MRE11 also acts downstream to degrade the newly-formed cruciform structures. Further studies are needed to address how precisely MRE11 engages during unperturbed S phase to facilitate fork reversal. We observed that fork slowing and reversal in MDC1-deficient cells occurs regardless of BRCA status, likely indicating that post replicative gaps that accumulate selectively in BRCA-deficient cells might not be the MRE11 primary substrate upstream of fork reversal. Recent works demonstrated that MRE11 acts during unperturbed DNA replication at heavily transcribed regions [43, 58]. Consistently, MDC1 has been recently linked to the stimulation of RNA-PolII transcription and pre-mRNA splicing [59]. These results suggest that, in absence of MDC1, altered transcription and splicing may trigger MRE11 activity with subsequent formation of ssDNA stretches required for fork reversal.

While extensive MRE11-mediated degradation of RVFs in BRCA1/2-defective cells has been previously associated with an increased sensitivity to chemotherapeutic agents [10, 11, 60], we show here that MRE11-mediated degradation in the context of MDC1 deficiency results in chemotherapy resistance. The amount of MRE11-mediated resection and its spatio-temporal dynamics may therefore be the critical determinant of PARPi and cisplatin response of BRCA-deficient tumors. Additionally, we demonstrated that EXO1-driven resection also contributes to fork slowing in *Mdc1* KO cells while DNA2 is not crucial for this process. These findings highlight the collaborative roles of MRE11 and EXO1 in DNA end resection, with MRE11 initiating the process and EXO1 further extending the resected gap. The complex nature of the nucleolytic processing at unchallenged or stalled RFs requires more research to be fully understood considering sensitization in PARPi-resistant HR-deficient tumors upon nuclease inhibitors as we recently observed in PARG-deficient cells [61].

Thus far, genetic alterations resulting in defective RF progression, stability or restart have usually been linked to a status of elevated genomic instability and increased sensitivity of cells to replication stress or DNA damage-inducing agents [30, 41, 43, 62–64]. In this study, we show that loss of MDC1 can also have the opposite effect. The delayed fork restart and reduced fork speed in MDC1-deficient cells does not stimulate the activation of global DDR signaling markers. In contrast, it results in an improved DNA damage tolerance and chromosomal stability in the context of BRCA1/2 deficiency. Interestingly, the function of MDC1 in modulating RF dynamics in the BRCA1/2-proficient RPE-1 cells does not seem to require an interaction with downstream repair proteins, such as 53BP1. It was recently reported that MDC1 enables the formation of 53BP1 nuclear bodies and promotes survival of cells following irradiation via its PST domain, even in the absence of the BRCT domains [21]. While the PST domain may be sufficient to enable the MDC1 protein to carry out these functions, our data indicates that it does not facilitate normal RF dynamics. Moreover, the TQXF motif, which is essential for efficient recruitment of downstream repair proteins including RNF8 and 53BP1, is not required for proper speed maintenance (Fig. S2D, F, G). However, this does not exclude the relevance of RNF8, RNF168 and 53BP1 for RF speed in other cellular contexts, as shown by Schmid et al. [30].

In summary, we show that MDC1 loss represents a novel mechanism of chemoresistance in BRCA1/2-deficient cells, which is independent of rewiring homology-directed DNA repair or restoration of RF protection. It is interesting to note that H2AX loss also increases chemotherapy resistance via a distinct mechanism, that is the stabilization of reversed RFs [10]. Although H2AX and MDC1 work together in DSB repair, our work points out that their roles in RF dynamics are separable. This discovery may contribute to our understanding of mechanisms of resistance that are independent of restoration of BRCA1/2 function. This is further supported by the finding of the group of Sharon Cantor and our data presented here, confirming the PARPi resistance when MDC1 was depleted in human MDA-MB-436 (*BRCA1*-mutated) [53] and PEO1 (*BRCA2*-mutated) cancer cells. It may therefore be useful to check the role of remodeling MDC1 status and RF progression in second biopsies derived from tumors of patients who acquired drug resistance.

## Supplementary information

Supplementary Figures

## Data Availability

The authors declare that all data supporting the findings of this study are available within the article or from the corresponding author upon request. Sequencing of the CRISPR/Cas9 genetic screens was performed at the Netherlands Cancer Institute. The results of the MAGeCK analysis are available in Table S1. Raw sequencing data of the genetic screen are available in European Nucleotide Archive (ENA) under accession number PRJEB74933.
