# Supplementary material for: MDC1 counteracts replication fork reversal and mediates chemosensitivity in BRCA1/2-deficient tumors
Source: Oncogene. 2025 Dec 17;45(4):491–504. doi: 10.1038/s41388-025-03659-8 (PMC12815688; doi:10.1038/s41388-025-03659-8)
Supplement: Supplementary file 1 — Supplementary Figures [file 41388_2025_3659_MOESM1_ESM.pdf]

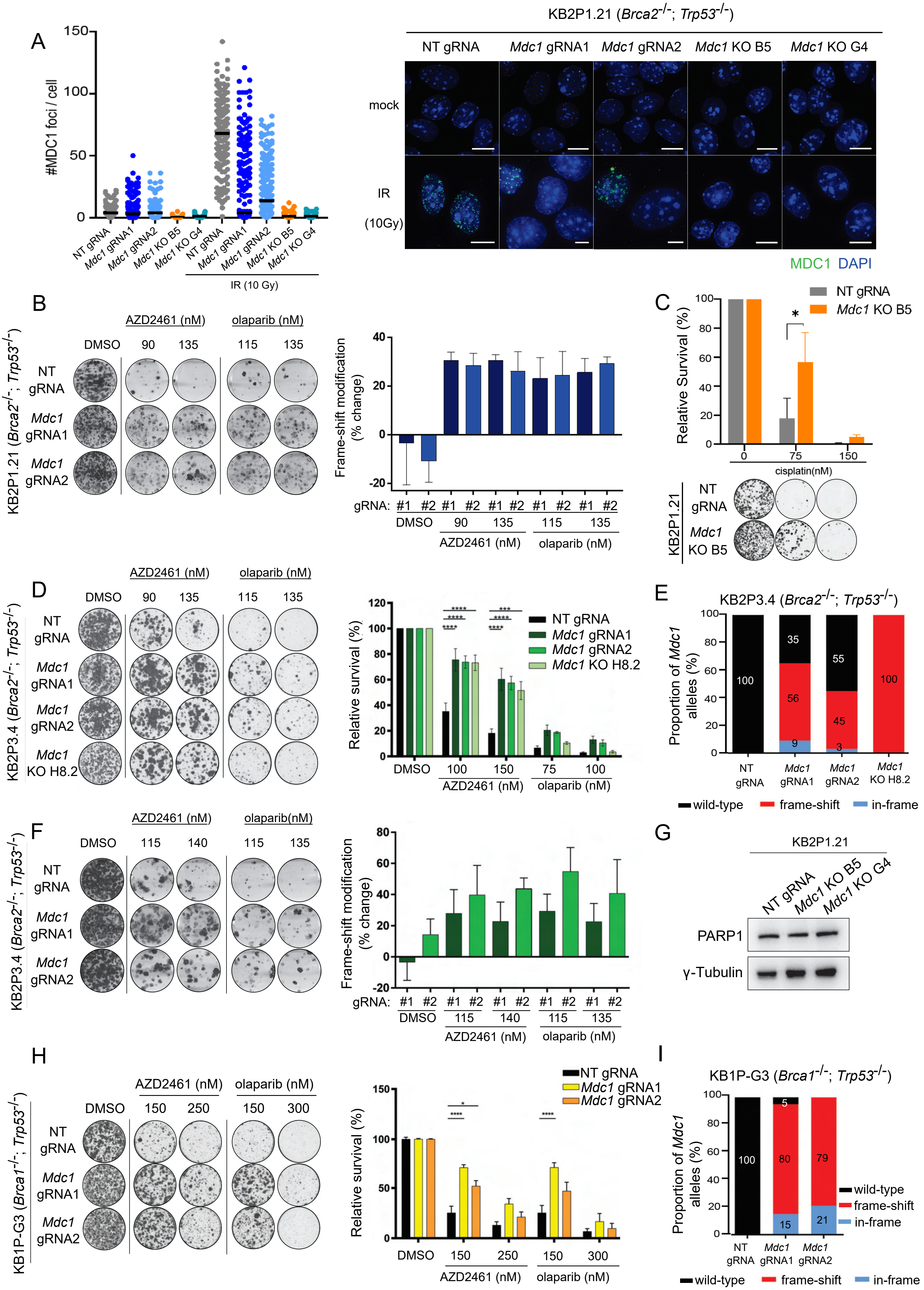

A
#MDC1 foci / cell
150
100
50
0
NT gRNA
Mdc1 gRNA1
Mdc1 gRNA2
Mdc1 KO B5
Mdc1 KO G4
IR (10 Gy)
KB2P1.21 (Brca2-/-; Trp53-/-)
NT gRNA
Mdc1 gRNA1
Mdc1 gRNA2
Mdc1 KO B5
Mdc1 KO G4
mock
IR
(10Gy)
MDC1 DAPI
B
AZD2461 (nM)
olaparib (nM)
DMSO
90
135
115
135
NT gRNA
Mdc1 gRNA1
Mdc1 gRNA2
KB2P1.21 (Brca2-/-; Trp53-/-)
Frame-shift modification (% change)
40
20
0
-20
gRNA: #1 #2 #1 #2 #1 #2 #1 #2 #1 #2
DMSO
90
135
115
135
AZD2461 (nM)
olaparib (nM)
C
NT gRNA
Mdc1 KO B5
Relative Survival (%)
100
80
60
40
20
0
*
0
75
150
cisplatin(nM)
KB2P1.21
NT gRNA
Mdc1 KO B5
D
AZD2461 (nM)
olaparib (nM)
DMSO
90
135
115
135
NT gRNA
Mdc1 gRNA1
Mdc1 gRNA2
Mdc1 KO H8.2
KB2P3.4 (Brca2-/-; Trp53-/-)
Relative survival (%)
120
100
80
60
40
20
0
****
***
NT gRNA
Mdc1 gRNA1
Mdc1 gRNA2
Mdc1 KO H8.2
DMSO
100
150
75
100
AZD2461 (nM)
olaparib (nM)
E
KB2P3.4 (Brca2-/-; Trp53-/-)
Proportion of Mdc1 alleles (%)
100
80
60
40
20
0
100
35
56
9
55
45
3
100
NT gRNA
Mdc1 gRNA1
Mdc1 gRNA2
Mdc1 KO H8.2
wild-type
frame-shift
in-frame
F
AZD2461 (nM)
olaparib(nM)
DMSO
115
140
115
135
NT gRNA
Mdc1 gRNA1
Mdc1 gRNA2
KB2P3.4 (Brca2-/-; Trp53-/-)
Frame-shift modification (% change)
80
60
40
20
0
-20
gRNA: #1 #2 #1 #2 #1 #2 #1 #2 #1 #2
DMSO
115
140
115
135
AZD2461 (nM)
olaparib (nM)
G
KB2P1.21
NT gRNA
Mdc1 KO B5
Mdc1 KO G4
PARP1
γ-Tubulin
H
AZD2461 (nM)
olaparib (nM)
DMSO
150
250
150
300
NT gRNA
Mdc1 gRNA1
Mdc1 gRNA2
KB1P-G3 (Brca1-/-; Trp53-/-)
NT gRNA
Mdc1 gRNA1
Mdc1 gRNA2
Relative survival (%)
150
100
50
0
*
****
DMSO
150
250
150
300
AZD2461 (nM)
olaparib (nM)
I
KB1P-G3 (Brca1-/-; Trp53-/-)
Proportion of Mdc1 alleles (%)
100
80
60
40
20
0
100
5
80
15
79
21
wild-type
frame-shift
in-frame
NT gRNA
Mdc1 gRNA1
Mdc1 gRNA2
wild-type
frame-shift
in-frame

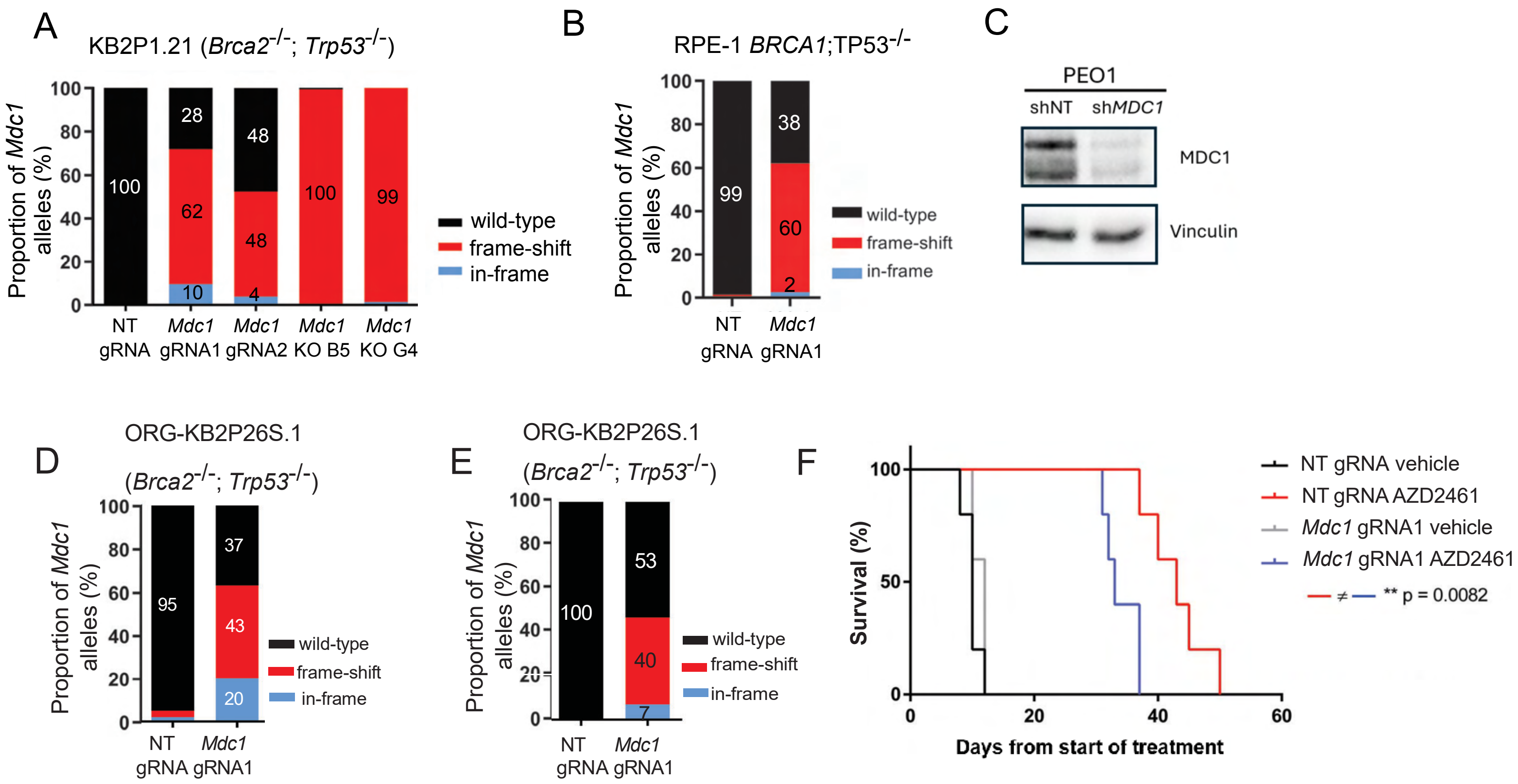
A
KB2P1.21 (Brca2-/-; Trp53-/-)
Proportion of Mdc1 alleles (%)
100
28
62
10
48
48
4
100
99
NT gRNA
Mdc1 gRNA1
Mdc1 gRNA2
Mdc1 KO B5
Mdc1 KO G4
wild-type
frame-shift
in-frame
B
RPE-1 BRCA1;TP53-/-
99
38
60
2
NT gRNA
Mdc1 gRNA1
C
PEO1
shNT
shMDC1
MDC1
Vinculin
D
ORG-KB2P26S.1
(Brca2-/-; Trp53-/-)
95
37
43
20
E
ORG-KB2P26S.1
(Brca2-/-; Trp53-/-)
100
53
40
7
F
Survival (%)
Days from start of treatment
NT gRNA vehicle
NT gRNA AZD2461
Mdc1 gRNA1 vehicle
Mdc1 gRNA1 AZD2461
≠ ** p = 0.0082

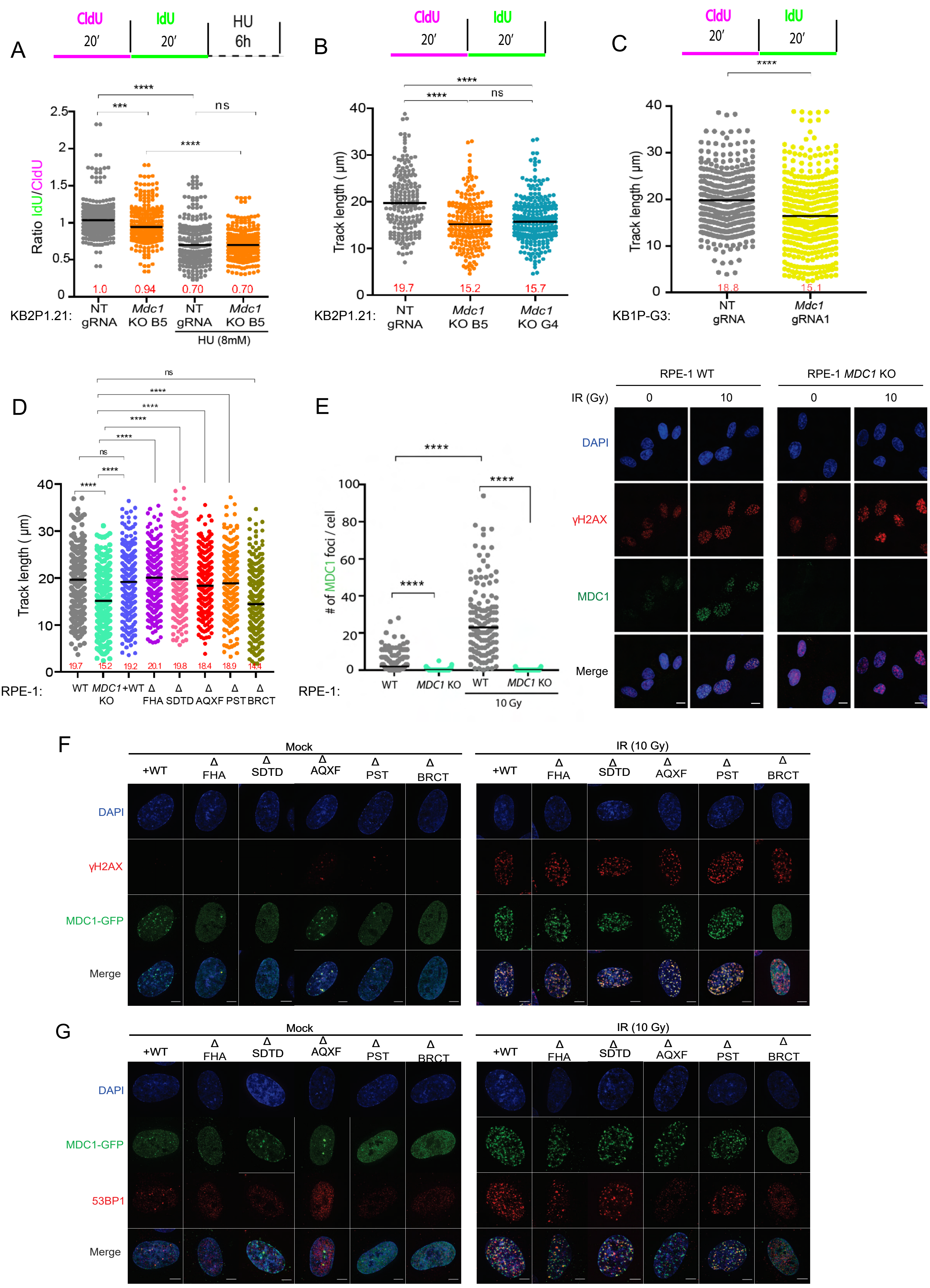

A
CldU 20′
IdU 20′
HU 6h
Ratio IdU/CldU
1.0
0.94
0.70
0.70
KB2P1.21:
NT gRNA
Mdc1 KO B5
NT gRNA
Mdc1 KO B5
HU (8mM)
B
CldU 20′
IdU 20′
Track length (μm)
19.7
15.2
15.7
KB2P1.21:
NT gRNA
Mdc1 KO B5
Mdc1 KO G4
C
CldU 20′
IdU 20′
Track length (μm)
18.8
15.1
KB1P-G3:
NT gRNA
Mdc1 gRNA1
D
Track length (μm)
19.7
15.2
19.2
20.1
19.8
18.4
18.9
14.4
RPE-1:
WT
MDC1 KO
+WT
Δ FHA
Δ SDTD
Δ AQXF
Δ PST
Δ BRCT
E
# of MDC1 foci / cell
RPE-1:
WT
MDC1 KO
WT
MDC1 KO
10 Gy
RPE-1 WT
RPE-1 MDC1 KO
IR (Gy)
0
10
0
10
DAPI
γH2AX
MDC1
Merge
F
Mock
IR (10 Gy)
+WT
Δ FHA
Δ SDTD
Δ AQXF
Δ PST
Δ BRCT
DAPI
γH2AX
MDC1-GFP
Merge
G
Mock
IR (10 Gy)
+WT
Δ FHA
Δ SDTD
Δ AQXF
Δ PST
Δ BRCT
DAPI
MDC1-GFP
53BP1
Merge

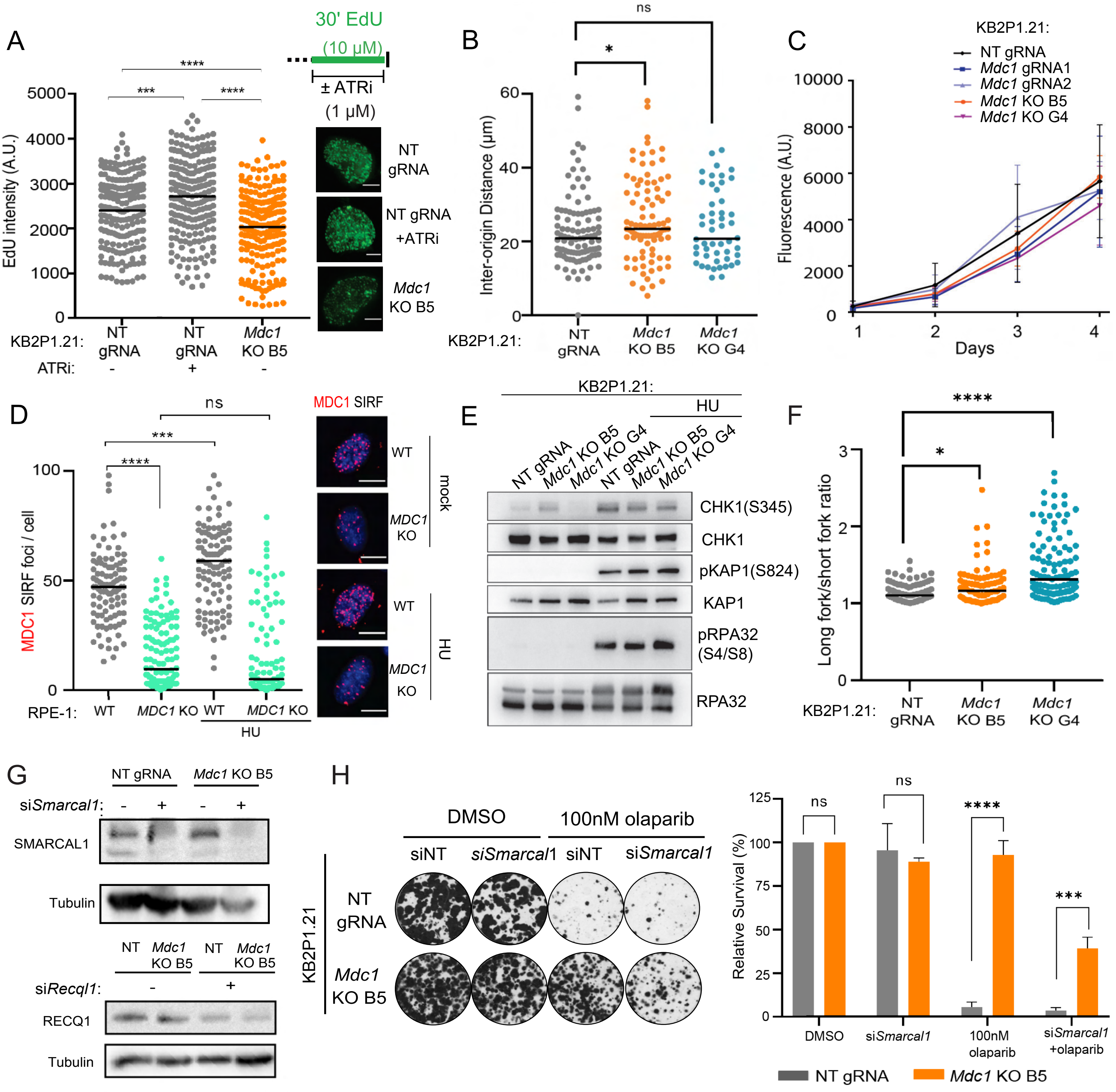
A
30' EdU
(10 μM)
± ATRi
(1 μM)
****
***
****
EdU intensity (A.U.)
5000
4000
3000
2000
1000
0
KB2P1.21:
NT gRNA
NT gRNA
Mdc1 KO B5
ATRi:
-
+
-
NT gRNA
NT gRNA +ATRi
Mdc1 KO B5
B
ns
*
Inter-origin Distance (μm)
60
40
20
0
KB2P1.21:
NT gRNA
Mdc1 KO B5
Mdc1 KO G4
C
KB2P1.21:
NT gRNA
Mdc1 gRNA1
Mdc1 gRNA2
Mdc1 KO B5
Mdc1 KO G4
Fluorescence (A.U.)
10000
8000
6000
4000
2000
0
1
2
3
4
Days
D
ns
***
****
MDC1 SIRF foci / cell
100
50
0
RPE-1:
WT
MDC1 KO
WT
MDC1 KO
HU
MDC1 SIRF
WT
MDC1 KO
WT
MDC1 KO
mock
HU
E
KB2P1.21:
HU
NT gRNA
Mdc1 KO B5
Mdc1 KO G4
NT gRNA
Mdc1 KO B5
Mdc1 KO G4
CHK1(S345)
CHK1
pKAP1(S824)
KAP1
pRPA32 (S4/S8)
RPA32
F
****
*
Long fork/short fork ratio
3
2
1
0
KB2P1.21:
NT gRNA
Mdc1 KO B5
Mdc1 KO G4
G
NT gRNA
Mdc1 KO B5
siSmarcal1:
-
+
-
+
SMARCAL1
Tubulin
NT
Mdc1 KO B5
NT
Mdc1 KO B5
siRecql1:
-
+
RECQ1
Tubulin
H
DMSO
100nM olaparib
siNT
siSmarcal1
siNT
siSmarcal1
KB2P1.21
NT gRNA
Mdc1 KO B5
ns
ns
****
***
Relative Survival (%)
125
100
75
50
25
0
DMSO
siSmarcal1
100nM olaparib
siSmarcal1 +olaparib
NT gRNA
Mdc1 KO B5

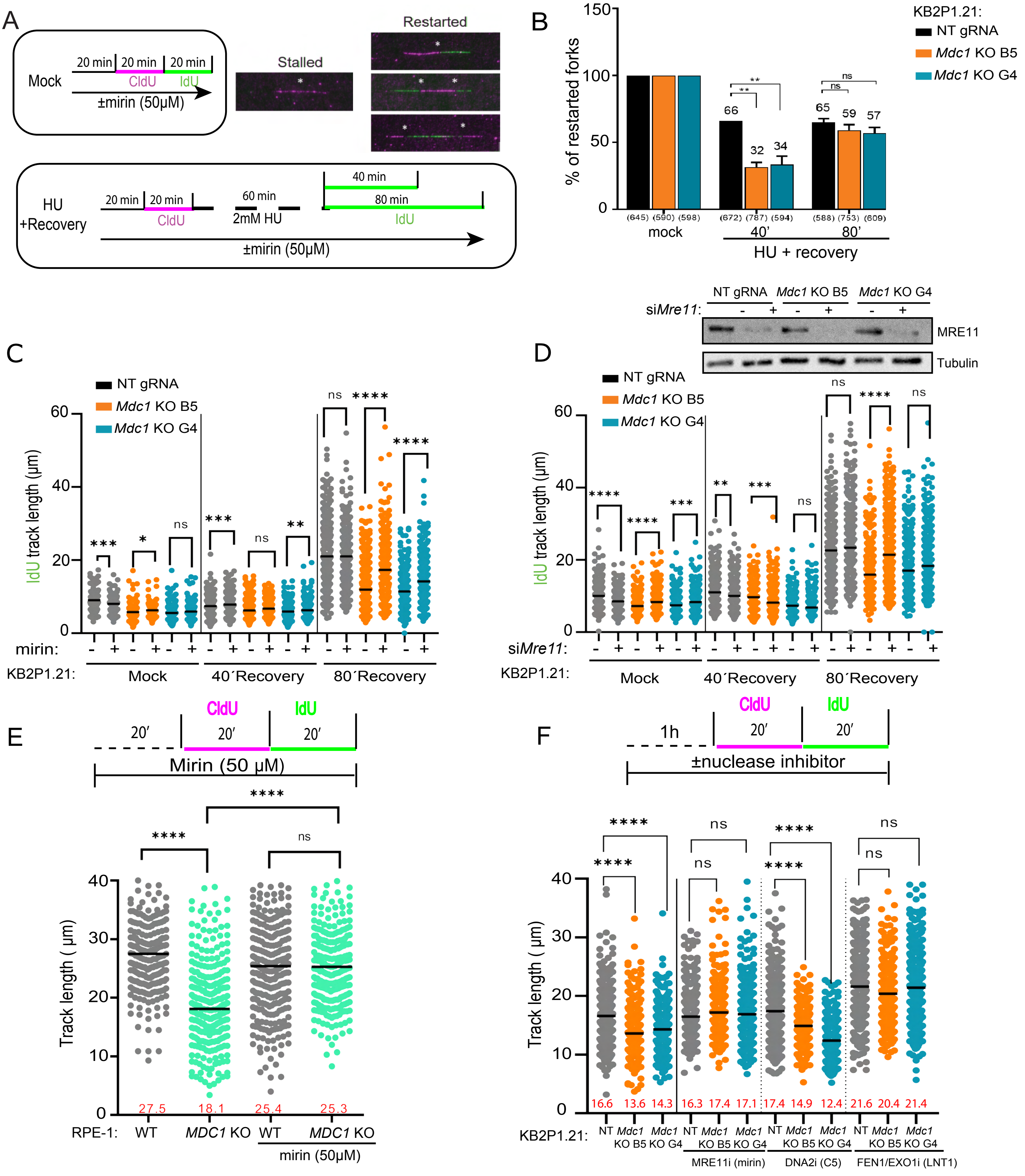
A
Mock
20 min
20 min
20 min
CldU
IdU
±mirin (50μM)
Stalled
Restarted
HU
+Recovery
20 min
20 min
60 min
40 min
80 min
CldU
2mM HU
IdU
±mirin (50μM)
B
% of restarted forks
150
100
50
0
KB2P1.21:
NT gRNA
Mdc1 KO B5
Mdc1 KO G4
66
32
34
65
59
57
**
**
ns
ns
(645) (590) (598)
(672) (787) (594)
(588) (753) (609)
mock
40'
80'
HU + recovery
C
NT gRNA
Mdc1 KO B5
Mdc1 KO G4
IdU track length (μm)
60
40
20
0
***
*
ns
***
ns
**
ns
****
****
mirin:
KB2P1.21:
Mock
40´Recovery
80´Recovery
D
NT gRNA
Mdc1 KO B5
Mdc1 KO G4
siMre11:
MRE11
Tubulin
IdU track length (μm)
****
****
***
**
***
ns
ns
****
ns
KB2P1.21:
Mock
40´Recovery
80´Recovery
E
20'
CldU
20'
IdU
20'
Mirin (50 μM)
****
****
ns
Track length (μm)
40
30
20
10
0
27.5
18.1
25.4
25.3
RPE-1:
WT
MDC1 KO
WT
MDC1 KO
mirin (50μM)
F
1h
CldU
20'
IdU
20'
±nuclease inhibitor
****
****
ns
ns
ns
****
****
ns
ns
Track length (μm)
16.6
13.6
14.3
16.3
17.4
17.1
17.4
14.9
12.4
21.6
20.4
21.4
KB2P1.21:
NT
Mdc1 KO B5
Mdc1 KO G4
MRE11i (mirin)
DNA2i (C5)
FEN1/EXO1i (LNT1)

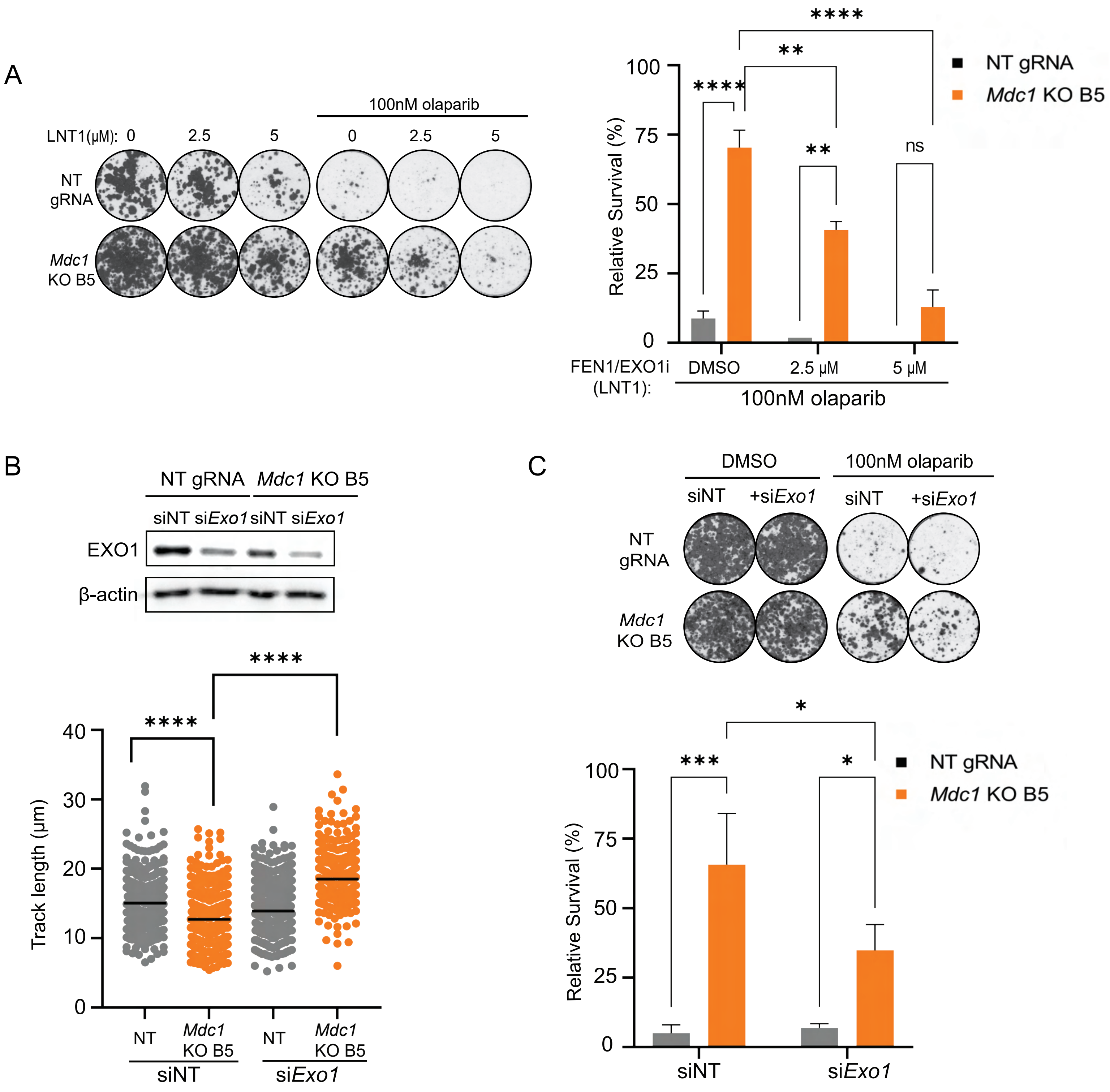

A
100nM olaparib
LNT1(μM): 0
2.5
5
0
2.5
5
NT gRNA
Mdc1 KO B5
****
**
****
**
ns
NT gRNA
Mdc1 KO B5
Relative Survival (%)
100
75
50
25
0
FEN1/EXO1i (LNT1):
DMSO
2.5 μM
5 μM
100nM olaparib
B
NT gRNA
Mdc1 KO B5
siNT siExo1 siNT siExo1
EXO1
β-actin
****
****
Track length (μm)
40
30
20
10
0
NT
Mdc1 KO B5
NT
Mdc1 KO B5
siNT
siExo1
C
DMSO
100nM olaparib
siNT
+siExo1
siNT
+siExo1
NT gRNA
Mdc1 KO B5
*
***
*
NT gRNA
Mdc1 KO B5
Relative Survival (%)
100
75
50
25
0
siNT
siExo1

id	num	neg|score	neg|p-value	neg|fdr	neg|rank	neg|goodsgrna	pos|score	pos|p-value	pos|fdr	pos|rank	pos|goodsgrna
**Ankmy2**	3	0.99862	0.9986	0.999895	21026	0	1.0883e-07	2.3509e-07	0.00495	1	3
**Mdc1**	3	0.94771	0.94754	0.999895	19773	0	5.7779e-07	2.1158e-06	0.022277	2	2
**Ric8**	3	0.99998	0.99998	0.999978	21058	0	2.1488e-05	5.4305e-05	0.352723	3	3
**Usp51**	3	0.9022	0.90183	0.999895	18836	0	2.5589e-05	6.7e-05	0.352723	4	2
**H2afx**	3	0.99996	0.99996	0.999978	21057	0	3.6542e-05	9.8032e-05	0.412871	5	3
**Ube3c**	3	0.99995	0.99995	0.999978	21056	0	4.6271e-05	0.00012812	0.44967	6	3
**Gm20854**	3	0.99994	0.99994	0.999978	21055	0	5.9992e-05	0.00017702	0.532532	7	3
**Cdrt4**	3	0.59565	0.68782	0.999895	14294	1	7.4532e-05	0.00021793	0.573639	8	2
**Lcn5**	3	0.70296	0.7458	0.999895	15551	1	0.00010477	0.00031902	0.746425	9	2
**Sprr1b**	3	0.81303	0.8202	0.999895	17072	1	0.00012422	0.00037826	0.796535	10	2
**Brk1**	3	0.9909	0.99088	0.999895	20825	0	0.00014143	0.00042245	0.808731	11	3
**Olfr1276**	3	0.84304	0.8449	0.999895	17610	1	0.0001739	0.00051555	0.827905	12	1
**Tmem170**	3	0.99323	0.99319	0.999895	20869	0	0.00019774	0.00058184	0.827905	13	3
**Mrgprb2**	3	0.070688	0.15692	0.979018	3364	1	0.00022359	0.0006646	0.827905	14	2
**3830403N18Rik**	2	0.99977	0.99974	0.999933	21054	0	0.00023346	0.00068716	0.827905	15	2
**Wbscr27**	3	0.99973	0.99971	0.999933	21053	0	0.00026881	0.00077932	0.827905	16	3
**Palb2**	3	0.88625	0.88582	0.999895	18493	0	0.00027327	0.00079107	0.827905	17	1
**Trpc5**	3	0.98871	0.98872	0.999895	20760	0	0.00028127	0.00080565	0.827905	18	3
**Vmn1r60**	3	0.99967	0.99964	0.99993	21052	0	0.00032748	0.00092836	0.827905	19	3
**Clec4e**	3	0.99782	0.99779	0.999895	21002	0	0.00033109	0.00093824	0.827905	20	3

Supplementary Figure Legends

Supplementary Figure 1. MDC1 deficiency promotes PARPi resistance *in vitro*. A) Quantification of MDC1 foci formation in absence or presence of irradiation in KB2P1.21 cells. Median of measured values in three independent experiments is shown. Representative immunofluorescence images are shown at the right. Scale bars represent 10 µm. **B)** Competition assay in KB2P1.21 polyclonal cell lines with representative images (left) and quantification of change in the frequency of frame-shift modifications following the treatment with with two PARP inhibitors(right). Mean ± SD of three independent experiments. **C)** Representative images and quantification of clonogenic assays with KB2P1.21 cells treated with cisplatin or DMSO. The data represent mean ± SEM of three independent experiments**.** Two-way ANOVA test; ns= non-significant, *p<0.01. **D)** Representative images and quantification of clonogenic assays with KB2P3.4 cells treated with olaparib or DMSO. The data represent mean ± SEM of three independent experiments**.** Two-way ANOVA test; ***p<0.001, ****p<0.0001. **E)** TIDE analysis demonstrating the *Mdc1*-targeting efficiency in KB2P3.4 cells. **F)** Competition assay in polyclonal KB2P3.4 cell lines with representative images (left) and quantification of change in the frequency of frame-shift modifications following the treatment with PARPi (right). Mean ± SD of three independent experiments. **G)** Western blotting of PARP1 and γ-Tubulin in NT gRNA and *Mdc1* KO B5 and G4 cells. Similar results were observed in three independent experiments. **H)** Clonogenic survival assay of KB1P-G3 cells expressing NT, or *Mdc1*-targeting gRNAs upon treatment with PARP inhibitors. Representative images (left) and quantification (right) are shown. Mean ± SEM of at least three independent experiments is shown. Two-way ANOVA test; *p <0.05, ****p<0.0001. **I)** TIDE analysis showing the frequency of WT and modified *Mdc1* alleles in KB1P-G3 cells.

Supplementary Figure 2. MDC1 deficiency confers PARPi resistance *in vitro and in vivo.* **A)** TIDE analysis showing the modification rate of the *Mdc1* gene in the KB2P1.21 cells expressing non-targeting (NT) or *Mdc1*-targeting gRNA. **B)** TIDE analysis showing the modification rate of the *MDC1* gene in the RPE-1 cells expressing non-targeting (NT) or *MDC1*-targeting gRNA. **C)** Western blotting of MDC1 and vinculin in NT shRNA or *MDC1*-targeting shRNA treated PEO1 cells. **D)** TIDE analysis of KB2P 3D organoid lines used in the *in vivo* experiment using olaparib treatment. **E)** TIDE analysis of KB2P 3D organoid lines used in the in vivo experiment using AZD2461 treatment. **F)** Kaplan-Meier curve showing the overall survival of the vehicle- or AZD2461-treated mice. *P*-value was calculated with the Mantel-Cox test.

Supplementary Figure 3. MDC1 does not restore stability of replication forks but regulates fork progression. A) DNA fiber assay showing RF degradation in KB2P1.21 cells upon HU-induced RF stalling. Minimum of 120 forks per sample were analyzed and the IdU/CldU ratio was calculated. Median of values measured from three independent experiments is shown. Mann-Whitney test; ns= non-significant, ***p<0.001, ****p<0.0001. **B)** DNA fiber assay showing reduced RF speed in KB2P1.21 *Mdc1* knockout lines B5 and G4. Track lengths of at least 100 forks were measured. Median of track lengths is shown. Similar results were observed in at least three biological replicates. Mann-Whitney test; ns= non-significant, ****p<0.0001. **C)** Track lengths analysis in KB1P-G3 cells expressing NT, or *Mdc1*-targeting gRNA. Minimum of 100 forks per sample were analyzed and median of values measured from three independent experiments is shown. Mann-Whitney test; ****p<0.0001. **D)** DNA fiber assay in RPE-1 cells expressing various MDC1 domain-lacking mutants. The median of track lengths from three independent experiments is shown. Mann-Whitney test; ns= non-significant, ****p<0.0001. **E)** Immunofluorescence analysis showing the loss of MDC1 IRIF formation in RPE-1 *MDC1* knockout cell line. The scale bar represents 10 µm. Data was collected form three independent experiments. Mann-Whitney

test; ****p<0.0001. **F)** Representative images from immunofluorescence staining demonstrating the ability of the MDC1 WT/mutant constructs expressed in RPE-1 cells to localize to DSB sites upon irradiation. The scale bars represent 5 μm. **G)** Representative images from immunofluorescence staining performed to assess the ability of the MDC1 WT/mutant constructs to recruit 53BP1 to the DSB sites. The scale bars represent 5 μm.

Supplementary Figure 4. MDC1 is associated with active replication forks and regulates their speed and fork reversal independently of new origin firing and changes in cell proliferation rate. A) Imaging-based analysis of EdU incorporation upon release of new origins by ATR inhibitor AZ20 or upon *Mdc1* KO in KB2P1.21 cells. The scale bars represent 5 μm. The results were obtained from three independent experiments. Mann-Whitney test; ns= non-significant, ***p<0.001, ****p<0.0001. **B)** Inter-origin distance scored in KB2P1.21 cells based on six independent experiments. Mann-Whitney test; ns= non-significant, *p <0.05. **C)** Proliferation rate of KB2P1.21 cells expressing NT gRNA, two *Mdc1*-targeting gRNAs and two KO cell lines B5 and G4. Mean ± SD of at least three biological replicates is shown. **D)** Analysis of MDC1 localization at active replication forks by SIRF. Representative images and quantification are shown. Similar results were obtained from at least three independent experiments. Scale bars represent 5 μm. One-way Anova test; ns= non-significant, ***p<0.001, ****p<0.0001. **E)** Western blotting of DDR markers before and after 1 mM HU for 24 h in NT gRNA, B5 and G4 KB2P1.21 cells. Similar results were obtained from three independent experiments. **F)** Fork asymmetry rates scored in KB2P1.21 cells based on six independent experiments. Mann-Whitney test; ns= non-significant, *p <0.05, ****p<0.0001. **G)** Efficiency of *Smarcal1* knockdown (top) and *Recql1* knockdown (bottom) was confirmed in western blot. **H)** Representative images and quantification of clonogenic assay with siRNA-mediated *Smarcal1* knockdown on KB2P1.21 cells after treatment with olaparib. The data represent mean ± SEM of three independent experiments**.** Two-way ANOVA test; *p <0.05, ***p<0.001, ****p<0.0001.

Supplementary Figure 5. MDC1 loss leads to delayed fork restart due to MRE11- and EXO1-dependent nascent DNA shortening. A) Scheme of RF restart experiments and examples of stalled or restarted forks, the asterisks show examples of the quantified events. **B)** Analysis of the percentage of restarted forks without treatment or after RF stalling with HU and two recovery time-points. Mean ± SD of three independent experiments is shown. Two-way ANOVA test; ns= non-significant, **p<0.01. **C)** IdU track length analysis of the restarted forks without and mirin treatment. Median of values from three independent experiments is shown. Mann-Whitney test; ns= non-significant, p <0.05, **p <0.01, ***p<0.001, ****p<0.0001. **D)** IdU track length analysis of the restarted forks without and after *Mre11*-targeting siRNA treatment. Efficiency of *Mre11* knockdown was confirmed in western blot (top). Median of values from three independent experiments is shown (bottom). Mann-Whitney test; ns= non-significant, *p <0.05, **p <0.01, ***p<0.001, ****p<0.0001. **E)** Total track length in RPE-1 cells without and after treatment with 50 μM mirin. Median of three independent experiments is shown. Mann-Whitney test; ns= non-significant, ****p<0.0001. **F)** Total track length in KB2P1.21 cells without and after treatment with 50 μM MRE11i (mirin), 20 μM DNA2i (C5) or 10 μM EXO1/FEN1i (LNT1). Median of three independent experiments is shown. Mann-Whitney test; ns= non-significant, ****p<0.0001.

Supplementary Figure 6. MDC1 regulates replication fork speed and PARPi sensitivity by suppressing EXO1 activity. A) Representative images and quantification of clonogenic assay with KB2P1.21 cells showing treatment response to EXO1i/FEN1i LNT1 or olaparib alone, and to a combined treatment. Mean ± SD of three independent experiments is shown. Two-way ANOVA test; ns= non-significant, **p <0.01, ****p<0.0001 **B)** Efficiency of *Exo1* knockdown was confirmed in western blot (top). Total track length in KB2P1.21 cells without and after *Exo1*-targeting siRNA treatment. Mann-Whitney test; ****p<0.0001. **C)** Representative images and quantification of clonogenic assay with siRNA-mediated *Exo1* knockdown on KB2P1.21 cells after treatment with olaparib. Mean ± SD of three

independent experiments is shown. Two-way ANOVA test; ns= non-significant, *$p <0.05$, ***$p<0.001$.

Table S1. Ranked list of the highest-scoring gene candidates. Candidate genes were identified based on 6 biological replicates of the CRISPR/Cas9-based PARPi resistance screen.
